# Deep sequencing reveals exceptional diversity and modes of transmission for bacterial sponge symbionts

**DOI:** 10.1111/j.1462-2920.2009.02065.x

**Published:** 2010-08

**Authors:** Nicole S Webster, Michael W Taylor, Faris Behnam, Sebastian Lücker, Thomas Rattei, Stephen Whalan, Matthias Horn, Michael Wagner

**Affiliations:** 1 Australian Institute of Marine Science, PMB 3, Townsville Mail CentreQld 4810, Australia; 2 School of Biological Sciences, University of AucklandPrivate Bag 92019, Auckland, New Zealand; 3 Department of Microbial Ecology, University of ViennaAlthanstr. 14, A-1090 Vienna, Austria; 4 Department of Genome Oriented Bioinformatics, Technische Universität MünchenAm Forum 1, 85354 Freising, Germany

## Abstract

Marine sponges contain complex bacterial communities of considerable ecological and biotechnological importance, with many of these organisms postulated to be specific to sponge hosts. Testing this hypothesis in light of the recent discovery of the rare microbial biosphere, we investigated three Australian sponges by massively parallel 16S rRNA gene tag pyrosequencing. Here we show bacterial diversity that is unparalleled in an invertebrate host, with more than 250 000 sponge-derived sequence tags being assigned to 23 bacterial phyla and revealing up to 2996 operational taxonomic units (95% sequence similarity) per sponge species. Of the 33 previously described ‘sponge-specific’ clusters that were detected in this study, 48% were found exclusively in adults and larvae – implying vertical transmission of these groups. The remaining taxa, including ‘*Poribacteria*’, were also found at very low abundance among the 135 000 tags retrieved from surrounding seawater. Thus, members of the rare seawater biosphere may serve as seed organisms for widely occurring symbiont populations in sponges and their host association might have evolved much more recently than previously thought.

## Introduction

Marine sponges (phylum *Porifera*) are among the most ancient of the extant metazoans, and have sparked recent interest due to their ecological importance and production of a diverse range of pharmacologically active metabolites (Vogel, 2008). They also play host to extraordinarily dense and diverse microbial communities, which comprise up to 40% of sponge volume and contribute to many aspects of sponge biology, including carbon and nitrogen nutrition and chemical defence (Hentschel *et al.*, 2006; Taylor *et al.*, 2007a). As perhaps the oldest of all symbioses between microbes and Metazoa, these associations provide a window to early animal evolution (Taylor *et al.*, 2007b). However, many fundamental questions relating to sponge symbiont ecology and evolution have remained unanswered due to constraints associated with currently applied techniques (e.g. lack of sequencing depth in sponge symbiont diversity surveys).

Twenty bacterial phyla and both major lineages of *Archaea* have been reported from sponges (Taylor *et al.*, 2007a; Webster *et al.*, 2008; Zhu *et al.*, 2008), yet it is the apparent specificity of these symbionts that is most intriguing. Molecular surveys based on the 16S rRNA gene suggest that many of the known sponge-associated microbes occur only within sponges (Hentschel *et al.*, 2002; Taylor *et al.*, 2007a). The presence of these microorganisms in distantly related sponges from geographically disparate regions, coupled with their apparent absence from seawater or other hosts, has raised questions about how these associations evolved and are maintained. Answers to other fundamental issues relating to sponge–microbe associations have also proven elusive. For example, what is the magnitude of sponge microbiome diversity? How does this diversity vary in different sponge species within the same environment? How do sponges acquire the majority of their symbionts (vertically or environmentally)? Documented vertical transmission of diverse microbial assemblages has highlighted a potential mechanism for maintaining these symbioses (Schmitt *et al.*, 2007; 2008; Sharp *et al.*, 2007; Steger *et al.*, 2008), and deep sequencing will elucidate the role of the rare seawater biosphere in environmental transmission of sponge symbionts.

Here we applied the recently developed, massively parallel bacterial 16S rRNA gene tag pyrosequencing approach (Sogin *et al.*, 2006) to the marine sponge species *Ianthella basta*, *Ircinia ramosa* and *Rhopaloeides odorabile* from Australia's Great Barrier Reef and to surrounding seawater samples to fully explore the magnitude of sponge microbiome diversity and enhance our understanding of the modes of symbiont acquisition.

## Results and discussion

### Extraordinary diversity of sponge symbiont communities

**Fig. 1:**
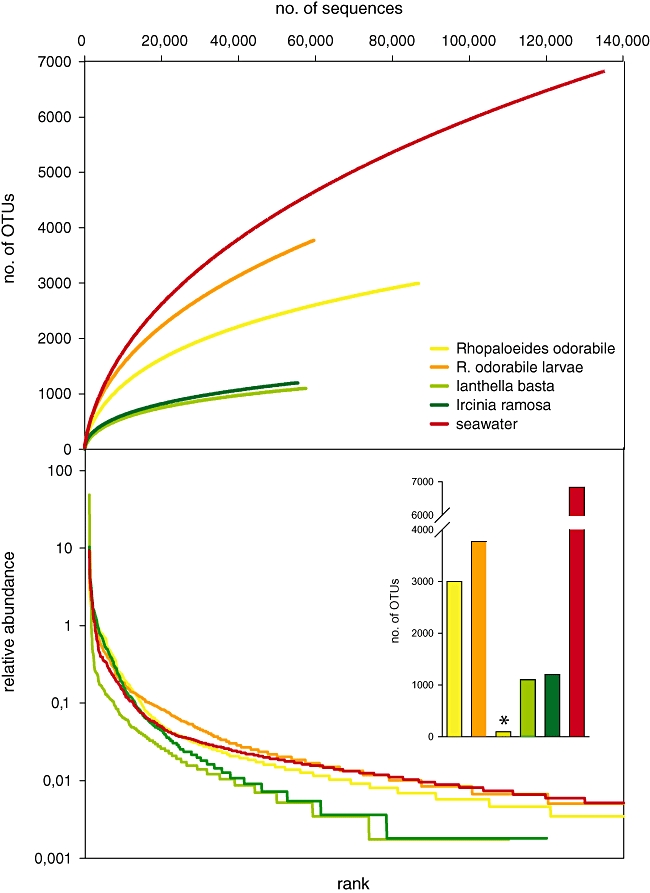
Diversity of sponge-associated bacterial communities and bacteria in the surrounding seawater. Rarefaction curves (top) and rank-abundance curves (bottom; only the first 1400 ranks are displayed) based on bacterial operational taxonomic units (OTUs) at a 95% sequence similarity threshold. Inset shows the number of OTUs detected in this study and in all publicly available 16S rRNA gene clones from *R. odorabile*, which contain the V6 region (*n* = 313; labelled with ‘*’).

Approximately 259 000 bacterial 16S rRNA-V6 tag sequences (between 50 and 60 nucleotides in length) were obtained from the marine sponges *I. basta*, *I. ramosa* and *R. odorabile*. The *R. odorabile* samples included both adult females and larvae, and for all sponge samples three biological replicates were analysed. Prior to this study, fewer than 600 16S rRNA gene sequences had been reported from a single sponge species (Enticknap *et al.*, 2006; Mohamed *et al.*, 2008). In addition, 135 000 tags were sequenced from four replicate samples of the surrounding seawater. In contrast to previous studies, diversity coverage was high for all sponge samples, with rarefaction curves approaching asymptotes in most cases (Fig. 1and Fig. S1). The total number of detected operational taxonomic units (OTUs) at 95% sequence similarity was much higher in all three adult sponge species than what has previously been reported for any sponge: 1099, 1199 and 2996 in *I. basta*, *I. ramosa* and *R. odorabile* respectively. However, the microbial communities in the three sponges were still less diverse than those in the surrounding seawater, which contained more than 6800 95%-level OTUs (Fig. 1). The exceptionally high diversity of the sponge-associated bacterial communities revealed here is also reflected in the Chao1 and ACE richness estimates, which at the 95% sequence similarity level predicted 1547/1498 OTUs for *I. basta*, 1707/965 OTUs for *I. ramosa*, and 4543/4352 OTUs for *R. odorabile*. With up to 3000 detected, and more than 4500 estimated, symbiont taxa per sponge species, these marine metazoa contain by far the highest number of different bacterial symbionts documented for an invertebrate host. For example, 943 clone sequences from the coral *Montastraea faveolata* contained 178 distinct OTUs (97% similarity threshold) with Chao1 estimates of a total of 307 ribotypes (Sunagawa *et al.*, 2009). The termite *Reticulitermes speratus* houses some 367 bacterial OTUs (97% similarity threshold) within its gut (Hongoh *et al.*, 2005). Symbiont richness in sponges is comparable to the number of different microbes in the human gut system. Examination of 11 831 16S rRNA genes cloned from the human gut yielded less than 400 bacterial OTUs (using a 99% sequence similarity threshold) (Eckburg *et al.*, 2005). Total OTU richness in that study was estimated at 500, while a total of 1200 ‘species’ have now been recorded from various human gut studies (Zoetendal *et al.*, 2008), and two recent studies indicated even higher numbers (Dethlefsen *et al.*, 2008; Peterson *et al.*, 2008). However, it should be noted that due to the heterogeneity of the 16S rRNA sequence data in these symbiont census studies the respective OTU calculations are based on different fragments of the 16S rRNA gene with different conservation profiles.

As expected for very diverse communities (Hughes *et al.*, 2001), all samples contained a relatively low proportion of highly abundant bacteria, with the bulk of the diversity composed of rare organisms represented by only one or a few sequence tags (Fig. 1). Of particular note was the sponge *I. basta*, in which a single gammaproteobacterial genus-level OTU comprised 49% of all sequence tags from this sponge. The V6 sequences within this OTU showed up to 96.7% sequence similarity to the sponge specific sequence clusters 70 and 72 (Taylor *et al.*, 2007a) as well as to a 16S rRNA sequence of an endosymbiont of the beard worm *Oligobrachia mashikoi* (Kubota *et al.*, 2007).

### The sponge microbiome is species-specific

**Fig. 2:**
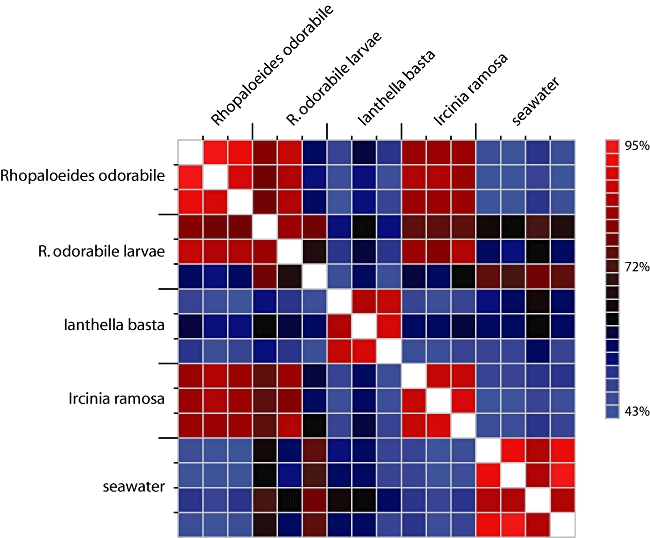
Similarity between sponge-associated bacterial communities and bacteria in the surrounding seawater. A heatmap illustrating Bray–Curtis similarities based on taxonomic assignments of V6 sequence tags of each replicate sponge (*n* = 3 per species) and seawater (*n* = 4) samples at the class level (80% sequence similarity) is shown.

Taxonomic V6-tag assignments revealed that replicates of the same sample type contained similar microbial communities, irrespective of the phylogenetic level examined (Fig. 2, Fig. S2), demonstrating (i) that different individuals of the same sponge species from the same environment harbour similar microbiomes, and (ii) that different sponge species from the same environment possess distinct symbiont communities, although the microbiomes of *R. odorabile* and *I. ramosa* showed some degree of similarity, particularly at the class level (Fig. 2). These results highlight the strong influence of the sponge host on the composition of its symbiont community and are consistent with previously published data obtained with molecular methods offering much lower diversity coverage (Taylor *et al.*, 2004; Webster *et al.*, 2004). An exception to the observed similarity between replicates in this study was the positioning of one *R. odorabile* larvae sample which, at some phylogenetic levels, showed similarity to the seawater samples (Fig. S2). This reflects contamination with seawater bacteria that were unavoidably cofiltered with the larvae during sampling and was evident to varying degrees in all larvae samples. Nevertheless, the three *R. odorabile* larvae samples contained the vast majority of microbial inhabitants detected in *R. odorabile* adults (79% of the assigned genera; Table S1). These results are consistent with Caribbean sponges where 28 different vertical transmission clusters belonging to at least 10 bacterial phyla and one archaeal phylum were detected in both adult and larval samples (Schmitt *et al.*, 2008). Overall, sponge-associated bacterial communities were clearly distinct from those in seawater, confirming earlier studies based on more limited techniques such as culturing (Santavy and Colwell, 1990) and denaturing gradient gel electrophoresis (Taylor *et al.*, 2005).

### Community composition of sponge-associated bacteria

**Fig. 3:**
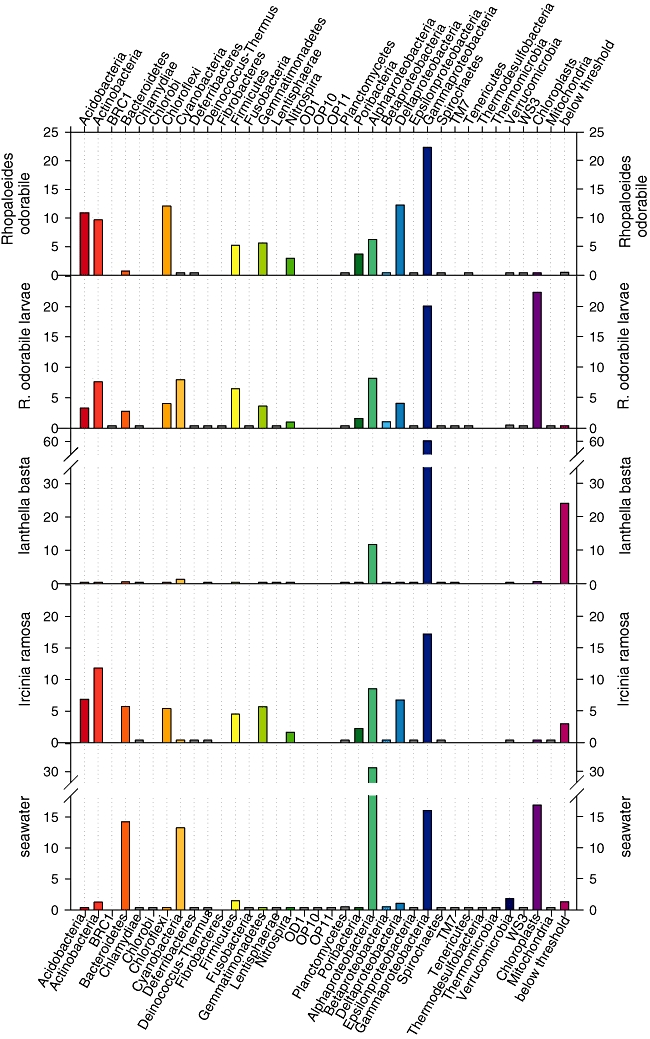
Taxonomic distribution of assigned V6 tag sequences. Bars represent for each sample the proportion (expressed as percentage) of tags that belong to a given bacterial phylum. The phylum *Proteobacteria* is split into the *Alpha*-, *Beta*-, *Delta*-, *Epsilon*- and *Gammaproteobacteria* classes. For clarity, values higher than 0 but below 0.4% are shown as 0.4% bars.

**Fig. 4:**
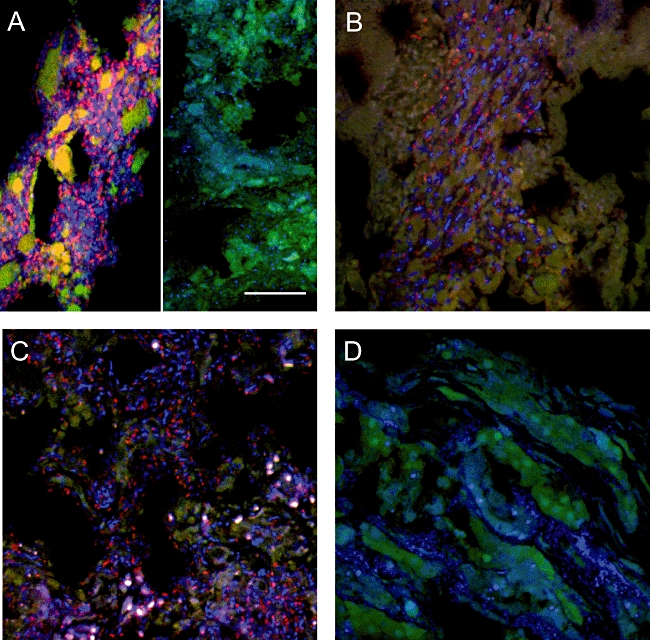
Detection of sponge-associated bacteria by fluorescence *in situ* hybridization (FISH). The presence of *Poribacteria* and nitrite-oxidizing bacteria of the genus *Nitrospira* in the three sponge species as well as in larvae of *R. odorabile* was confirmed using FISH probes. A. Left. Section of *R. odorabile* hybridized with a Cy3-labelled *Poribacteria*-specific probe (red) and a Cy5-labelled *Bacteria*-specific probe set (blue). Right. Section of *R. odorabile* hybridized with a Cy5-labelled *Nitrospira*-specific probe (blue). B. Section of an *R. odorabile* larvae hybridized with a Cy3-labelled *Nitrospira*-specific probe (red) and a Cy5-labelled *Poribacteria* -specific probe (blue). It should be noted that the detected bacteria are not located on the surface of the larvae and thus are no seawater contaminants. C. Section of *Ircinia ramonsa* hybridized with a Cy3-labelled *Nitrospira*-specific probe (red) and a Cy5-labelled *Poribacteria*-specific probe (blue). D. Section of *Ianthella basta* hybridized with a Cy5-labelled *Bacteria*-specific probe-set (blue). Consistent with the V6 16S rRNA gene tag analysis, the number of *Poribacteria* and *Nitrospira* were below the FISH detection limit in this sponge. Bar corresponds to 20 μm and applies to all figures.

It has been demonstrated that 16S rRNA hypervariable region tags, which are up to 15% divergent from their nearest reference match, provide equivalent taxonomic assignment to the respective full-length rRNA sequences (Huse *et al.*, 2008). In our study, 90% of all retrieved unique tags and 92% of all tags showed a similarity of at least 85% to reference sequences in the database and could thus be assigned with high accuracy. Of those sequence tags, which could be reliably assigned at phylum level (75% sequence similarity threshold), taxa containing common sponge symbionts such as the *Acidobacteria*, *Actinobacteria*, *Chloroflexi*, *Nitrospira* and *Proteobacteria* (especially the *Alpha-*, *Delta-* and *Gammaproteobacteria* classes) dominated in *R. odorabile* (adults and larvae) and *I. ramosa* (Fig. 3). The candidate phylum ‘*Poribacteria*’, until now found exclusively in sponges (Fieseler *et al.*, 2004; Taylor *et al.*, 2007a), was also abundant in these two hosts and this finding was confirmed by PCR-independent fluorescence *in situ* hybridization (Fig. 4). Additional sequences represented other groups with known sponge associates such as *Bacteroidetes*, *Cyanobacteria* and *Gemmatimonadetes*. The third sponge, *I. basta*, typically contained much lower abundances of these groups compared with the other sponges, though a high abundance of *Gamma-* and *Alphaproteobacteria* was evident. An abundance of *Cyanobacteria* and chloroplast sequences in seawater and *R. odorabile* larvae likely accounts for the aforementioned clustering together of these sample types in some analyses.

**Fig. 5:**
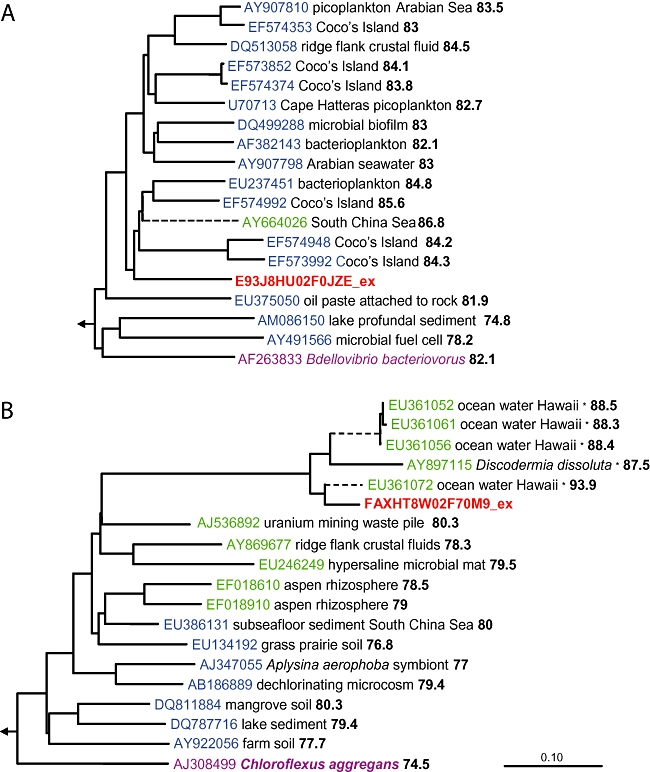
Phylogeny of PCR-extended V6-tags with low sequence similarities to published 16S rRNA sequences. Sequence similarities of the reference sequences to the extended V6 tag clone are indicated behind the annotation. Reference sequences are labelled in red, sequences included in the curated SILVA database used for inference of the tag affiliations are labelled in blue, sequences imported from public databases but not included in the curated SILVA database (e.g. because they were published recently or were too short) are labelled in green. Dashed lines connect short sequences which were added to the maximum likelihood tree via the ARB Parsimony Interactive Tool without changing the overall tree topology. Stars indicate that the respective sequence does not include the V6 region. The scale bar applies to both panels. A. Using the tag-specific PCR primer ATCACCGAGTTTCCTTAC, clone E93J8HU02F0JZE_ex (985 nucleotides in length) was amplified from one of the seawater replicate samples. This clone covers 44 bases (excluding the PCR primer binding region) of the targeted V6 tag sequence and all 44 nucleotides are identical to the original tag sequence. Phylogenetic analysis revealed that this clone is moderately related to the *Deltaproteobacteria* and has *Bdellovibrio bacteriovorus* as its closest cultured relative. All related sequences from the curated SILVA database had a similarity below 85.6%. B. Using the tag-specific PCR primer GGAGGTCCAGGCTATGTCA, clone FAXHT8W02F70M9_ex (997 nucleotides in length) was amplified from one of the adult *R. odorabile* samples. This product covers seven nucleotides (excluding the PCR primer target region) of the targeted V6 tag sequence and all seven nucleotides are identical to the original tag sequence. Phylogenetic analysis revealed that this clone is deeply branching within the *Chloroflexi* and has *Chloroflexus aggregans* as its closest cultured relative. All related sequences from the curated SILVA database had a similarity below 80.3%. It should be noted that the sequences most closely related to clone FAXHT8W02F70M9_ex are very short (< 886 nucleotides) and do not cover the V6 region.

A proportion of our tag sequences (4%) could not be assigned to known phyla because the similarity of the retrieved V6 tags to 16S rRNA sequences in the curated SILVA database was below 75% (Table S2). In order to check whether these non-assignable short V6 sequences indeed represent phylogenetic novelty not covered by the database, we used a novel approach whereby specific PCR primers targeting selected tag sequences were designed and applied together with a conserved *Bacteria* forward primer to amplify ∼1000 bp 16S rRNA gene fragments from the respective samples. In all cases, the retrieved 16S rRNA sequences had no close relatives in the database used for assignment and some of the amplified sequences showed similarities of below 85% to all publicly available 16S rRNA gene sequences. These findings demonstrate that at least some of the non-assignable tag sequences in fact represent previously unknown microbes with very low 16S rRNA sequence similarities (Hugenholtz *et al.*, 1998) (Fig. 5).

Unexpectedly, some sequence tags from the sponges were assigned to the phyla *Aquificae* and *Thermotogae* (Table S2), which contain hyperthermophilic microorganisms. As these tag sequences had similarities of only 78–84% to sequences in our reference database (Fig. S3), we again utilized the novel approach of PCR-mediated extension and subsequent phylogenetic analysis to evaluate their assignment. Specific PCR primers targeting the most abundant *Aquificae*- and *Thermotogae*-like tag sequences were designed and applied together with a conserved *Bacteria-*specific forward primer to amplify ∼1000 bp 16S rRNA gene fragments from the respective samples. Interestingly, the obtained 16S rRNA sequences had 100% sequence similarity to the respective tags, but in phylogenetic analysis unambiguously clustered with members of subdivision 4 of the phylum *Chloroflexi* (Fig. S3), which were not included in the database used for tag assignment because they had no taxonomic annotation in the original database. The incorrect assignment of some tags to the *Aquificae* and *Thermotogae* demonstrates that in some rare cases V6 tags representing lineages of a phylum not well represented in the reference database (as subdivision 4 of the *Chloroflexi* in our case) might be incorrectly assigned to other phyla despite application of a stringent assignment similarity threshold.

In addition to the *Aquificae* and *Thermotogae*, the tag sequencing performed during this study indicated the presence of six other phyla newly recorded at very low abundance from sponges, which would increase the number of bacterial phyla known from sponges from 20 to 26. The six phyla are BRC 1, *Deferribacteres*, *Fibrobacteres*, *Fusobacteria*, *Tenericutes* and WS3 (Fig. 3), although three of these phyla (BRC 1, *Fibrobacteres*, *Fusobacteria*) occurred only in the larvae samples and might thus originate from seawater. Except for 14 sequence tags (assigned to the *Fusobacteria*, the *Tenericutes* and WS3 respectively), the V6 sequence similarities to reference sequences from the database were below 85%. Thus, additional experiments would be required to unambiguously prove their presence in these metazoans. In this context it is interesting to note that a recent report claiming the presence of *Aquificae*, *Deferribacteres* and *Dictyoglomi* in the marine sponge *Aplysina fulva* (Hardoim *et al.*, 2009) was based on partial 16S rRNA gene sequence assignment using the RDP Classifier at low confidence limits. Our phylogenetic analyses of these sequences indicated that they are all in fact affiliated with the *Deltaproteobacteria*, which are well known from sponges (data not shown).

### Deep sequencing provides insights into bacterial function in sponges

For most of the detected sponge-associated taxa, metagenomic (Robidart *et al.*, 2008) and ecophysiological experiments (Wagner, 2009) will be required to make inferences about their function. Notable exceptions are those taxa involved in nitrification, for which a direct link between phylogeny and metabolism has been demonstrated (Bock and Wagner, 2007) and which are of functional importance for the ecology of sponges (Bayer *et al.*, 2008). In contrast to the sponge *Aplysina aerophoba* (Bayer *et al.*, 2008), no bacterial ammonia oxidizers were detected in the three sponges investigated in this study. This finding suggests that in these marine animals archaeal ammonia oxidizers dominate the first step in nitrification, consistent with the recently reported presence of crenarchaeal biomarkers in *R. odorabile* adults and larvae (Steger *et al.*, 2008) and in other sponges (Bock and Wagner, 2007; Hallam *et al.*, 2006; Meyer and Kuever, 2008; Hoffmann *et al.*, 2009). Interestingly, many V6 sequence tags assigned to the bacterial genus *Nitrospira* known to catalyse nitrite oxidation, the second step in nitrification, were present in *R. odorabile* larvae and adults as well as in *I. ramosa*, a result that was also confirmed by FISH analysis (Fig. 4). No other bacterial genera with recognized capability to oxidize nitrite were represented in notable numbers among the assigned tag sequences. In addition, in contrast to the sponge *Geodia barretti* (Hoffmann *et al.*, 2009), no indications for the presence of anaerobic ammonium oxidizers (ANAMMOX) were found in the three sponges investigated in this study.

In contrast to *Nitrospira*, other taxa [e.g. *Flavobacteriaceae*, *Synechococcus* (GpIIa) cyanobacteria and chloroplasts] were highly abundant in the seawater but relatively infrequent inside the sponges (Fig. 3, Table S3). This distribution pattern is typical for food bacteria, which were either present in the sponge canals during sampling or were in the process of being consumed by sponge cells. The latter process is very rapid and consumption of food bacteria by the sponge *Aplysina aerophoba* takes place within minutes of uptake by the sponge (Wehrl *et al.*, 2007). Consequently, classical 16S rRNA-based approaches have probably overlooked most of the food bacteria inside sponges, which remained detectable in this study by deep sequencing.

### Origin of sponge symbionts: vertical transmission and environmental acquisition from the rare seawater biosphere

A central question in contemporary sponge microbiology pertains to the origin of the so-called sponge-specific symbiont clusters. A given cluster, representing a monophyletic grouping of 16S rRNA gene sequences recovered exclusively from sponges, may contain > 50 sequences from > 20 geographically widespread host species (Taylor *et al.*, 2007a). How did these clusters evolve and are the respective symbionts indeed absent from seawater? Taxonomic radiation in sponges peaked during the Cambrian explosion (∼530 MYA) (Pisera, 2006), so if an individual sponge-specific lineage is derived from only one or a few colonization events, then these associations must be very ancient. Genomic evidence for microbial recognition mechanisms within the ancestral metazoan supports this theory (Taylor *et al.*, 2007b), although currently available data do not allow for a definitive answer. An alternative explanation for ‘sponge-specific’ clusters is that these microbes are in fact present in the surrounding seawater (providing a seed bank for colonization of sponges), but at such low abundances that they have not been detected in conventional molecular surveys of marine bacteria. A typical 16S rRNA gene-based diversity analysis involves sequencing of a few hundred clones, which is likely to be insufficient for detection of a particularly rare sequence in an environment with almost 7000 95%-OTUs (Fig. 1), while oceanic metagenome studies also tend to reveal only the more abundant community members. The superior sequencing depth offered by tag pyrosequencing provided the first opportunity to thoroughly explore the ‘rare biosphere’ (Sogin *et al.*, 2006) and systematically search for putatively sponge-specific bacterial taxa among the previously inaccessible fraction of oceanic microbial communities.

**Fig. 6:**
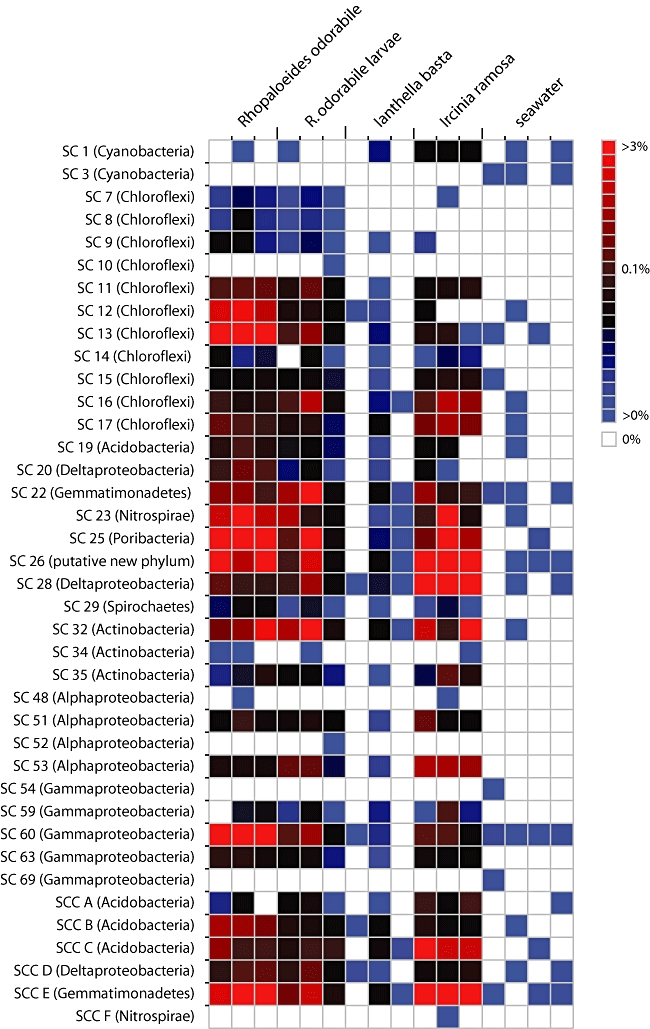
Occurrence of ‘sponge-specific’ 16S rRNA sequence clusters in sponge and seawater samples. Heatmap showing the distribution of representatives of previously described ‘sponge-specific’ 16S rRNA sequence clusters (Taylor *et al.*, 2007a) among the V6 tag sequences recovered in this study. Clusters with the prefix SC contain sequences previously reported only from sponges; the prefix SCC denotes clusters containing only sponge- and coral-derived sequences. The colour code indicates relative abundance, ranging from blue (low abundance) via black to red (high abundance); white indicates that no tag was assigned to the respective cluster. It should be noted that two 16S rRNA gene sequences affiliated with the *Poribacteria* were recently obtained in a metagenomic study of sea water sampled at several hundred metre depths (Pham *et al.*, 2008).

Many of our tag sequences (*n* = 52 270, or 13.3%) were found to represent previously described sponge-specific clusters (Taylor *et al.*, 2007a) (Fig. S4, Table S4), while clusters consisting exclusively of sponge- and coral-derived sequences comprised a further 3.6% of the data set. *Rhopaloeides odorabile* adults and larvae, as well as *I. ramosa*, contained the greatest proportion of sequences in sponge-specific clusters (32%, 16% and 28% respectively), while only few of these symbionts were recovered from *I. basta* (< 1% of all *I. basta*-derived tags; Fig. 6). However, it should be noted that the percentage of sponge-specific symbionts in *I. basta* might be much higher, as 49% of the tags from this sponge are highly similar to the gammaproteobacterial sponge-specific clusters 70 and 72 (which have identical V6 regions). Interestingly, some gammaproteobacterial 16S rRNA sequences not affiliated with clusters 70 and 72 also possess V6 regions with 100% sequence identity to some members of these clusters, and therefore an unambiguous assignment of V6 tags to these clusters is often impossible.

Seventeen of the 33 detected ‘sponge-specific’ sequence clusters were also detected in seawater samples, albeit in very low numbers and often in only a single replicate (Fig. 6). For five of these 17 ‘sponge-specific’ sequence clusters the seawater tags showed 100% sequence similarity with 16S rRNA sequences obtained from sponges. For most of the other 12 clusters, high similarities (above 90%) with reference sequences from sponges were also noticed. Our data suggest that those 48% of sponge-specific clusters, which were not detected among the 135 000 seawater-derived tag sequences, maintain their symbiotic association solely through vertical transmission, although the possibility that further sequencing of the rare seawater biosphere would reveal additional ‘sponge-specific’ clusters in this habitat cannot be ruled out. However, for the other ‘sponge-specific’ clusters, including abundant symbionts of presumed functional importance such as the ‘*Poribacteria*’ and lineages within the *Chloroflexi* and *Acidobacteria*, their unexpected detection in seawater either implies the existence of previously unknown, free-living non-symbiotic relatives or a lifestyle more complex than previously thought. These symbiont lineages are apparently extremely rare in seawater (and thus found only in some of the replicates from the environment) but highly enriched in sponge hosts (and often larvae), suggesting that the sponge may harvest these organisms from the rare biosphere in addition to their vertical transmission *via* the larvae (Fig. 6). A combination of vertical and horizontal transmission is required to maintain the association between the sponge *Ectyoplasia ferox* and its diverse sponge-specific microbes (Schmitt *et al.*, 2008). The establishment and maintenance of horizontally transmitted symbiosis has been extensively explored in the bobtail squid *Euprymna scolopes* and the luminous bacterium *Vibrio fischeri* (Nyholm and McFall-Ngai, 2004). In this example, the squid also acquires the symbiotic bacterium from a relatively low-abundance population (< 0.1%) that is resident in the seawater. In the squid-*Vibrio* model, the mechanisms for acquiring and maintaining the symbiosis have been fully elucidated as the association involves a single cultivable bacterium. In the case of sponges, which host diverse bacterial consortia, the mechanisms for symbiont acquisition and maintenance will be more difficult to ascertain. It is possible that the ‘sponge-specific’ sequence clusters detected in seawater may not have originated from viable cells or might be a result of symbiont release at the time of spawning or via damage/fragmentation of the adult tissue (Schmitt *et al.*, 2008). However, even in these scenarios, the released symbionts might serve as a source for re-infection of other sponges as per the squid-*Vibrio* model.

## Conclusions

16S rRNA gene tag pyrosequencing revealed an exceptionally high diversity of bacterial symbionts within three sponge species and suggested for several so-called sponge-specific symbiont clusters that the dual evolutionary modes of (i) vertical transmission and (ii) acquisition of symbionts from the (previously overlooked) rare seawater biosphere might operate together. This has important implications for our perception of the evolution of sponge–microbe associations, because it could explain the widespread distribution of these symbionts in many different sponge hosts without the need to postulate an ancient association.

## Experimental procedures

### Sample collection

The marine sponges *I. basta*, *I. ramosa* and *R. odorabile* were used in this study as they are common Great Barrier Reef species and occupy the same depth and habitat, allowing all samples to be collected within a 100 m^2^ area thereby avoiding confounding environmental variables. Sponge replicates were collected > 20 m apart although no minimum distance was used for collection of the different sponge species. Adult specimens were used for each of the three species and the larvae from *R. odorabile* were also collected. All sponge and larvae samples were collected by SCUBA from 15 m depth at Davies Reef, Queensland, Australia (19°09.35′S; 146°52.87′E) in January 2008. Samples were immediately frozen in liquid nitrogen for later DNA extraction or fixed in 4% paraformaldehyde (PFA) for FISH analysis. Larval *R. odorabile* specimens were collected by placing mesh traps over adult sponges between the hours of 1 pm and 4 pm, which is the period for larval release. *Rhopaloeides odorabile* larvae were then filtered onto sterile Kimwipes (Kimberley-Clark Professional, Milsons Pt Australia) and frozen in liquid nitrogen for DNA extraction. Due to extreme weather and unsafe conditions during larval collection, it was not possible to pre-filter or rinse the samples prior to freezing in liquid nitrogen. Additional larvae were transported back to the laboratory, individually rinsed in sterile seawater and fixed in 4% PFA for FISH. Larvae samples 4, 5 and 6 were obtained from female *R. odorabile* represented by samples 1, 2 and 3 respectively. Four replicate 1 l seawater samples were aseptically collected 10 m up-current of the sampled sponges (away from the coral bomby) and filtered through individual 0.2 μm sterivex filters (Durapore, Millipore, MA, USA), which were filled with 1.8 ml of lysis buffer [40 mM EDTA (pH 8.0), 50 mM Tris and 0.75 M sucrose] and frozen at −80°C.

### DNA extraction, PCR and sequencing

Frozen sponge tissue (approximately 0.5 g per sample) and pieces of Kimwipe containing approximately 20 *R. odorabile* larvae were aseptically transferred to 1.5 ml Eppendorf tubes using sterile scalpels and processed using two DNA extraction techniques. In the first method, grinding buffer (0.5 ml) was added to each replicate sample [100 mM Tris (pH 9.0), 100 mM EDTA (pH 8.0), 1% SDS and 100 mM NaCl]. Tubes were immersed in liquid nitrogen and ground with plastic pestles. Samples were incubated at 65°C for 60 min prior to addition of 187 μl 5 M potassium acetate. Samples were incubated on ice for 30 min and centrifuged at 8000 *g* for 15 min. The supernatants were transferred to fresh tubes and DNA was precipitated with 0.8 vol. of isopropanol. In the second method, samples were processed with a MO BIO PowerPlant DNA Isolation Kit as per the manufacturer's instructions (MO BIO Laboratories, CA, USA). DNA was extracted from seawater filters by addition of 200 μl lysozyme (10 mg ml^−1^), incubation at 37°C for 45 min, addition of 200 μl of proteinase K (0.2 μg ml^−1^) and 1% SDS and incubation at 55°C for 1 h. Lysates were recovered into fresh eppendorf tubes and DNA was extracted with a standard phenol:chloroform:isoamyl alcohol procedure and precipitated with 0.8 vol. of isopropanol. Equal volumes of DNA from each extraction method (20 ng total) from each sample were used to generate PCR amplicons (tags), which were pooled and sequenced by 454 pyrosequencing on a Roche GS20 system, as previously described by Huber and colleagues (2007). PCR and tag sequencing was performed at the Marine Biological Laboratory, Woods Hole, under the auspices of the International Census of Marine Microbes. In brief, several versions of the V6-flanking primers 967F and 1046R were used to maximize the diversity of amplified bacterial taxa (Huber *et al.*, 2007), and tag sequences which were less than 50 nt after removal of the amplification primers were flagged as low-quality and deleted. The tag sequence data set from this project is available at the VAMPS (Visualization and Analysis of Microbial Population Structures) website, which also provides helpful tools for data analysis and visualization (http://vamps.mbl.edu/). It should, however, be noted that the analyses presented in this study were performed on a custom made software platform and might thus differ in details from the VAMPS data.

### Taxonomic assignment of 16S rRNA V6 tag sequences

Taxonomic assignment of tag sequences was conducted using customized perl scripts and available software, similar to an approach described previously (Sogin *et al.*, 2006). First, each sequence was subjected to a blast (Altschul *et al.*, 1990) search against a manually curated SILVA 16S rRNA database (Pruesse *et al.*, 2007) (based on version 95, containing 193 262 bacterial sequences with taxonomic annotation at least at the phylum level). The 100 best hits were then aligned using muscle with parameters: -maxiters 2, -diags and -phyi (Edgar, 2004), and a Jukes-Cantor corrected distance matrix was constructed using phylip's dnadist (Felsenstein, 1989). The most similar sequence(s) (within a range of 0.1% sequence divergence compared with the most similar reference sequence) were then used for tag assignment according to the taxonomy of the Ribosomal Database Project (Cole *et al.*, 2007) and additional taxonomic information about sponge-specific clusters defined earlier (Taylor *et al.*, 2007a) (Fig. S4). In cases where the taxonomy of the most similar sequences was inconsistent, a majority rule was applied, and the tag was only assigned if at least 60% of all reference sequences shared the same taxonomic annotation (at the respective taxonomic level). To increase the accuracy of the assignments, different sequence similarity thresholds were used for different taxonomic levels, i.e. a tag was only assigned at the genus level if the sequence similarity to the most similar sequence(s) was above 95%. For assignment at the family, order, class and phylum level, 90%, 85%, 80% and 75% sequence similarity were used as thresholds respectively. For assignment to sponge-specific clusters or sponge-coral specific clusters a 75% sequence similarity threshold was applied. As the mutation rate within the V6 region is generally higher than the mean mutation rate of the 16S rRNA gene (Van de Peer *et al.*, 1996), our approach is very conservative and prevents over-assignment of sequence tags. Based on the tag assignment, the Bray–Curtis similarity between the analysed samples was calculated using the program PRIMER 5 (PRIMER-E, UK) and visualized as heatmaps using JColorGrid (Joachimiak *et al.*, 2006).

### Determination of OTUs and diversity estimates

To assign all 16S rRNA V6 tag sequences to OTUs, non-redundant data sets were constructed, in which each sequence tag occurred only once. These data sets were aligned using muscle with parameters: -maxiters 2, -diags and -phyi (Edgar, 2004), and a Jukes-Cantor corrected distance matrix was constructed using a newly developed Java program, which is optimized for the analysis of large multiple sequence alignments and requires significantly less calculation time and memory than phylip's dnadist (Felsenstein, 1989). For the calculation of distances, terminal gaps were ignored, and all other gaps were treated as single evolutionary events as described previously (Sogin *et al.*, 2006). The obtained distance matrix served as input for an enhanced version of dotur (Schloss and Handelsman, 2005), which mapped the non-redundant data set to the original data set in order to determine OTUs and to calculate rarefaction curves, rank-abundance plots and Chao1 richness estimates. Rarefaction curves and rank-abundance plots were visualized using SigmaPlot (Systat Software, USA).

### PCR-mediated extension of V6 tags

Specific PCR primers were designed for selected tag sequences assigned to the phyla *Aquificae* and *Thermotogae* and for selected tags with a similarity below 75% to 16S rRNA sequences in the curated SILVA database. These primers were used as reverse primers together with the *Bacteria*-specific forward primer 616V (Juretschko *et al.*, 1998) in order to PCR-amplify corresponding 16S rRNA gene fragments from the sponge or seawater samples. After cloning, sequencing and chimera checking with the program Pintail (Ashelford *et al.*, 2005) phylogenetic analysis was performed using RAxML (Stamatakis, 2006). The obtained 16S rRNA sequences were deposited at GenBank/EMBL/DDBJ under accession numbers GQ244304 to GQ244307.

### Fluorescence *in situ* hybridization

The presence of *Poribacteria* and nitrite-oxidizing bacteria of the genus *Nitrospira* in the three sponge species, as well as in larvae of *R. odorabile*, was confirmed by using previously published FISH probes and protocols (Amann *et al.*, 1990; Daims *et al.*, 1999; 2001; Fieseler *et al.*, 2004). FISH was performed with PFA-fixed cryosections of sponge material taken from the same individuals and collected larval biomass, respectively, which was also used for DNA extraction and subsequent 16S rRNA gene V6-tag analysis.

## Supporting information

**Fig. S1.** Diversity of sponge-associated bacterial communities and bacteria in the surrounding seawater.

**Fig. S2.** Similarity between sponge-associated bacterial communities and bacteria in the surrounding seawater.

**Fig. S3.** Maximum Likelihood-based phylogeny of PCR-extended V6-tags which were assigned to the phyla Aquificae and Thermotogae.

**Fig. S4.** Sponge-specific clusters identified in a previous analysis of all sponge-derived 16S rRNA gene sequences, which were publicly available as of 28 February 2006.

**Table S1.** Taxonomic assignments of V6 sequence tags from sponge and seawater samples at the genus level.

**Table S2.** Taxonomic assignments of V6 sequence tags from sponge and seawater samples at the phylum level.

**Table S3.** Taxonomic assignments of V6 sequence tags from sponge and seawater samples at the family level.

**Table S4.** Taxonomic assignments of V6 sequence tags from sponge and seawater samples to ‘sponge-specific’ 16S rRNA sequence clusters.
