# Supplementary material for: Deep sequencing reveals exceptional diversity and modes of transmission for bacterial sponge symbionts
Source: Environ Microbiol. 2010 Aug;12(8):2070–82. doi: 10.1111/j.1462-2920.2009.02065.x (PMC2936111; doi:10.1111/j.1462-2920.2009.02065.x)
Supplement: Supplementary file 1 [file emi0012-2070-SD1.pdf]

Deep sequencing reveals exceptional diversity and modes of transmission for marine bacterial sponge symbionts

Nicole S. Webster, Michael W. Taylor, Faris Behnam, Sebastian Lücker, Thomas Rattei, Stephen Whalan, Matthias Horn, Michael Wagner

Supporting Information

Supporting Figures **2**

Fig. S1 Diversity of sponge-associated bacterial communities and bacteria in the surrounding seawater. *2*

Fig. S2 Similarity between sponge-associated bacterial communities and bacteria in the surrounding seawater. *3*

Fig. S3 Maximum Likelihood-based phylogeny of PCR-extended V6-tags which were assigned to the phyla Aquificae and Thermotogae *4*

Fig. S4 Sponge-specific clusters identified in a previous analysis of all sponge-derived 16S rRNA gene sequences which were publicly available as of February 28, 2006. *5*

Supporting Tables **19**

Table S1 Taxonomic assignments of V6 sequence tags from sponge and seawater samples at the genus level *19*

Table S2 Taxonomic assignments of V6 sequence tags from sponge and seawater samples at the phylum level *32*

Table S3 Taxonomic assignments of V6 sequence tags from sponge and seawater samples at the family level *34*

Table S4 Taxonomic assignments of V6 sequence tags from sponge and seawater samples to 'sponge-specific' 16S rRNA sequence clusters *40*

Supporting Figures

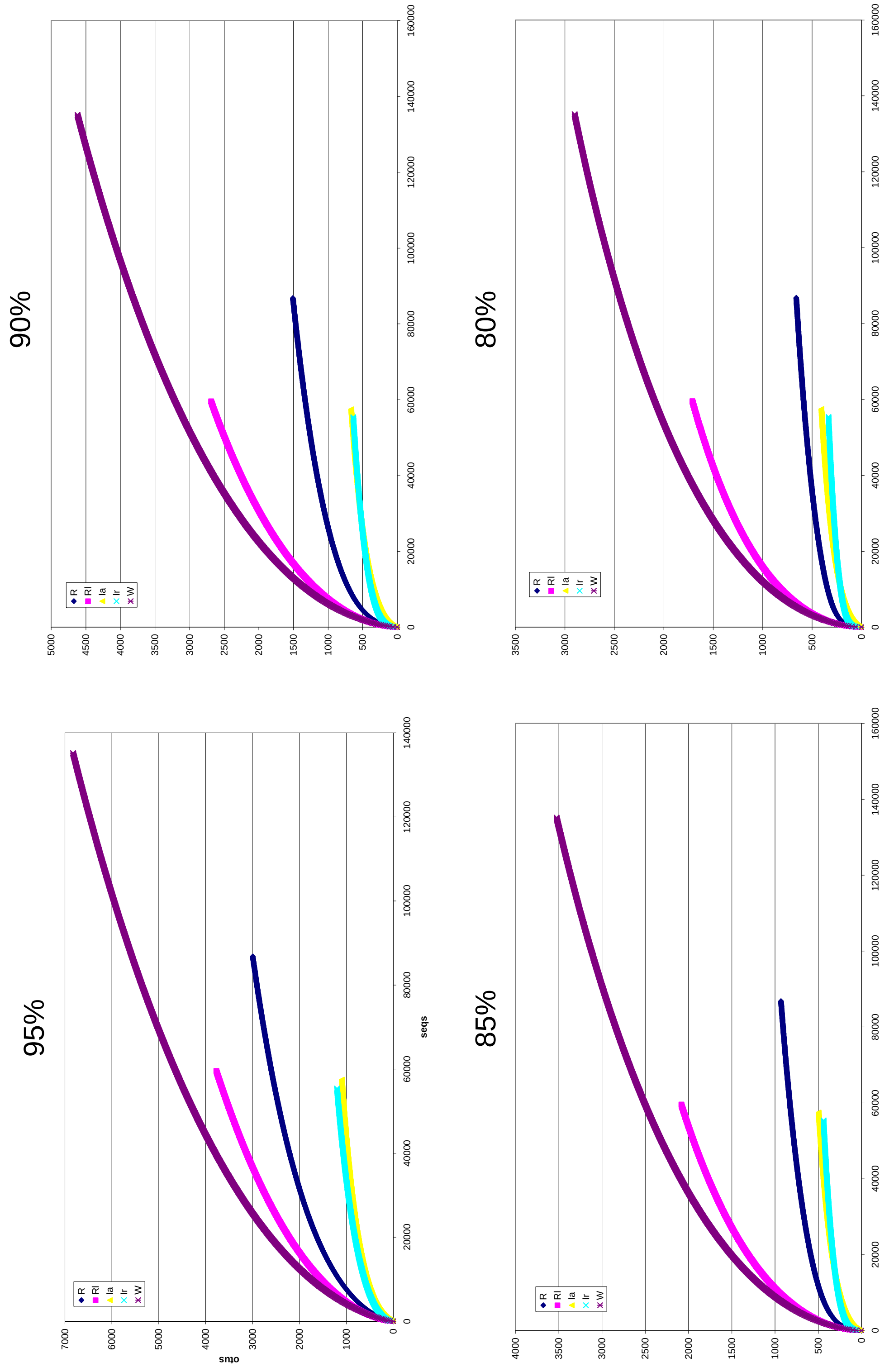

Fig. S1 **Diversity of sponge-associated bacterial communities and bacteria in the surrounding seawater.** Rarefaction curves showing the number of bacterial operational taxonomic units (OTUs) using 80%, 85%, 90%, and 95% sequence similarity thresholds for the sponges *Ianthella basta* (Ia), *Ircinia ramosa* (Ir), *Rhopaloeides odorabile* (adults (R) and larvae (Rl)), and surrounding seawater (W).

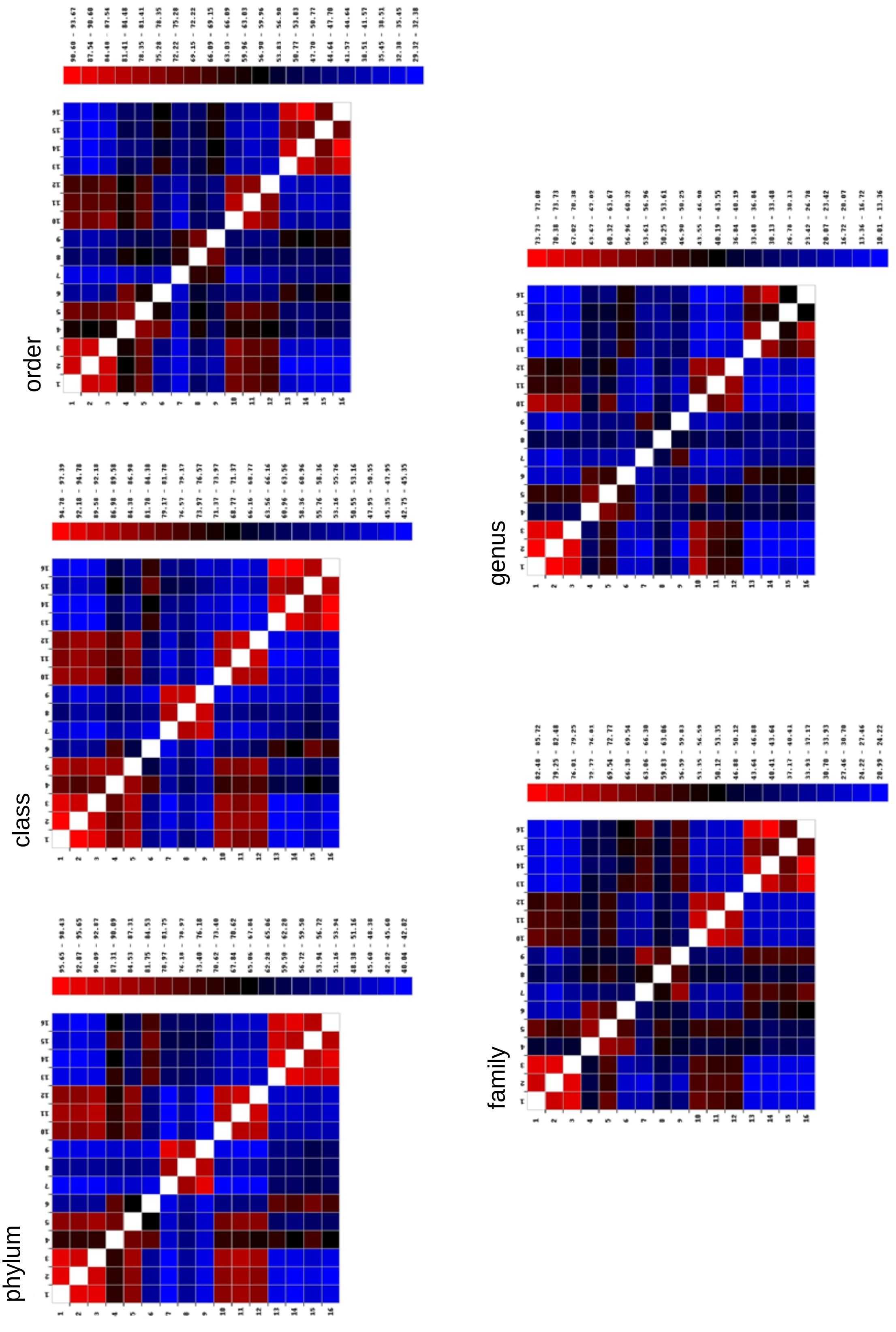

Fig. S2 **Similarity between sponge-associated bacterial communities and bacteria in the surrounding seawater.** Heatmaps illustrating Bray-Curtis similarities based on taxonomic assignments of V6 sequence tags of sponge and seawater samples at different taxonomic levels. Sample numbers: 1-3, *R. odorabile*; 4-6, *R. odorabile* larvae; 7-9, *I. basta*; 10-12, *I. ramosa*; 13-16, seawater.

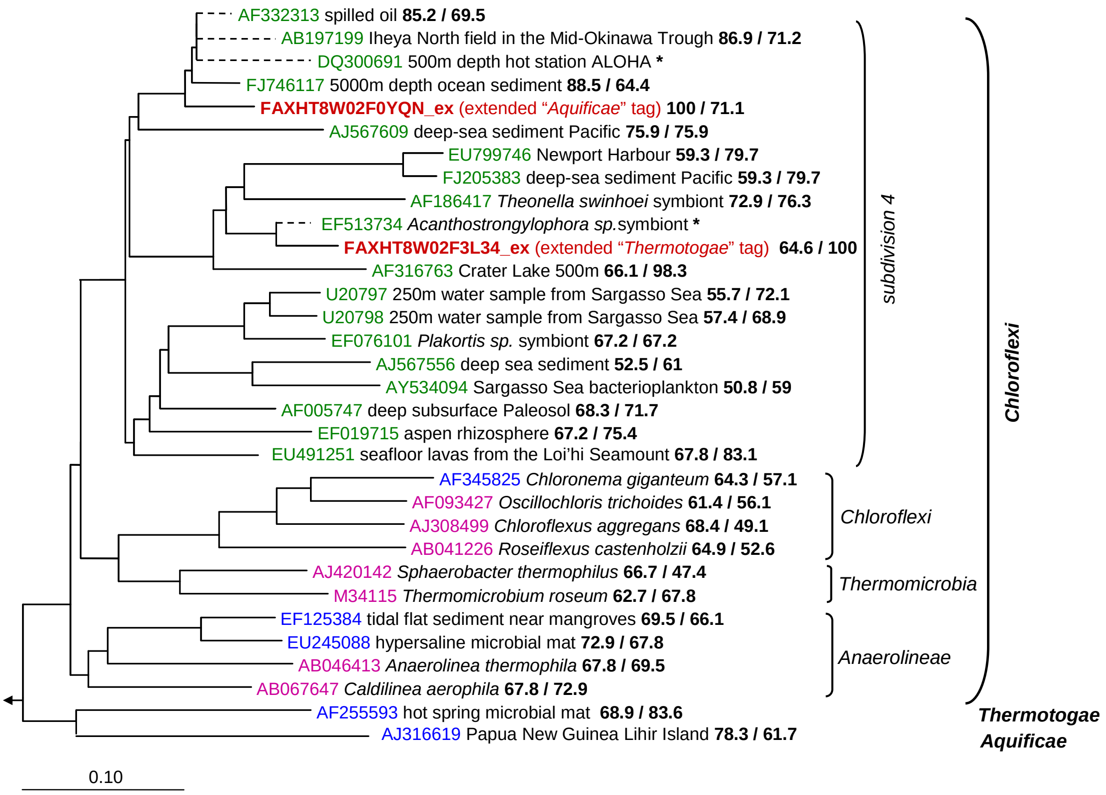

Fig. S3. **Maximum likelihood-based phylogeny of PCR-extended V6-tags which were assigned to the phyla *Aquificae* and *Thermotogae*.** Specific PCR primers were designed for the most frequently retrieved tag sequence for each of these phyla and these primers were used together with a *Bacteria*-specific forward primer to PCR-amplify the corresponding 16S rRNA gene fragments from the sponge symbionts of *R. odorabile*. Similarities of the extended tag sequences and the reference sequences to the respective V6 tag sequences are indicated in bold behind the sequence names. Sequences included in the database used for taxonomic tag assignment are labelled in blue, sequences imported from public databases but not included in the assignment database (e.g. because they were published recently or were too short) are labelled in green. Dashed lines connect short sequences which were added to the maximum likelihood tree via the ARB Parsimony Interactive Tool without changing the overall tree topology. Stars indicate that the respective sequence does not include the V6 region. Using the *Aquificae*-tag-specific PCR primer GGGTCACCCTGGCTTTC, clone FAXHT8W02F0YQN_ex (1001 nucleotides) was amplified. This clone covers 29 nucleotides (excluding the PCR primer binding region) of the targeted V6 tag sequence and this sequence stretch is identical to the respective tag sequence. Using the *Thermotogae*-tag-specific PCR primer TTGCGGGTCCCTTGCCTT, clone FAXHT8W02F3L34_ex (1016 nucleotides) was amplified. This clone covers 31 nucleotides (excluding the PCR primer target region) of the targeted V6 tag sequence and all 31 nucleotides are identical to the original tag sequence. Phylogenetic analysis revealed that both extended V6 clones cluster within the phylum *Chloroflexi*.

Fig. S4 **Sponge-specific clusters identified in a previous analysis of all sponge-derived 16S rRNA gene sequences which were publicly available as of February 28, 2006.** Cluster labels have been superimposed on the original figures with the following nomenclature: SC=sponge-specific cluster; SCC=sponge + coral-specific cluster. Figures are reproduced from the original article (Taylor, M.W., Radax, R., Steger, D., Wagner, M. 2007. Sponge-associated microorganisms: evolution, ecology and biotechnological potential. Microbiol. Mol. Biol. Rev. 71: 295-347) with generous permission of the American Society for Microbiology.

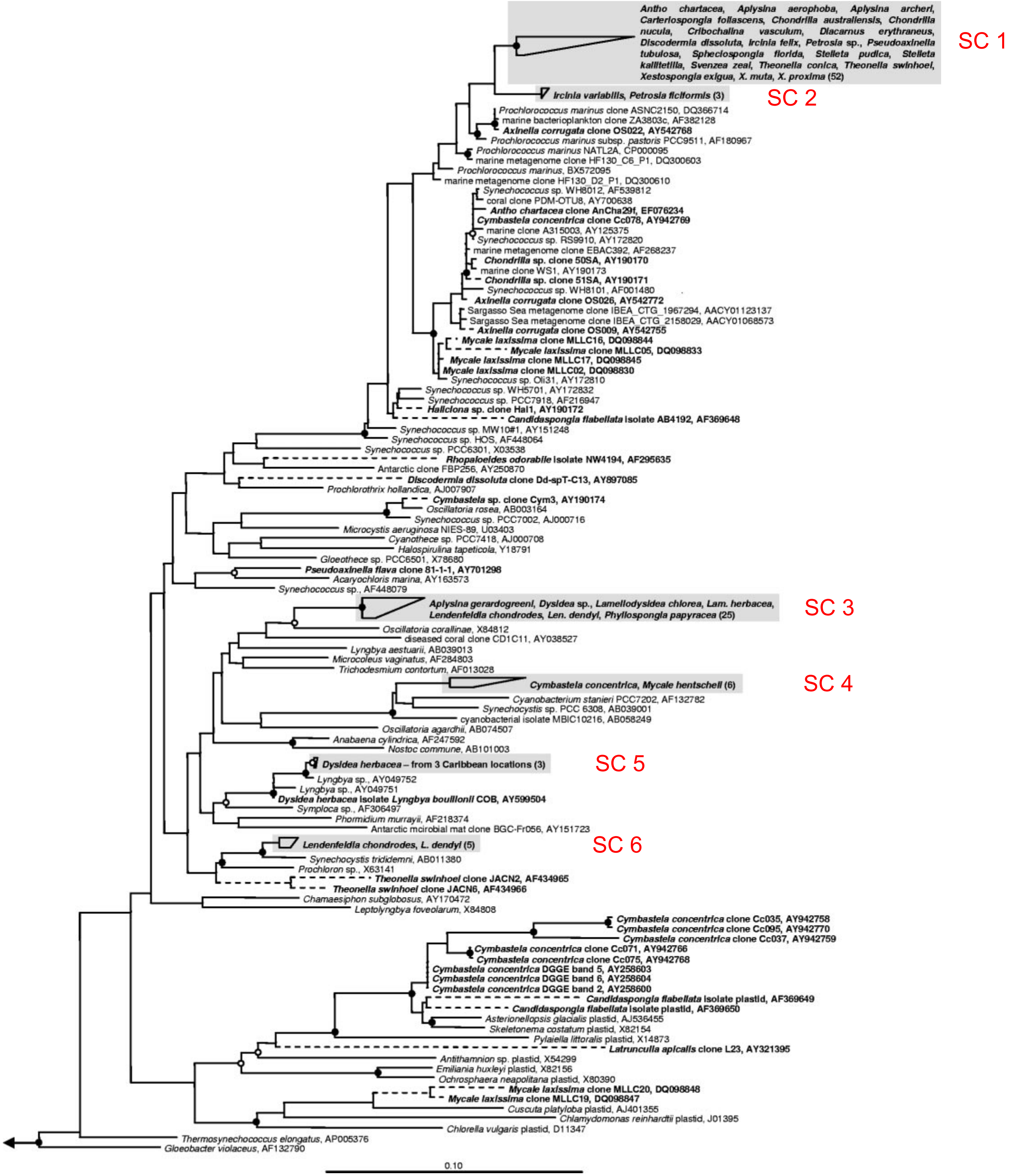

Sponge-specific clusters within the *Cyanobacteria*, based on Fig. 5 from Taylor *et al.* (2007). Reproduced with kind permission of the American Society for Microbiology.

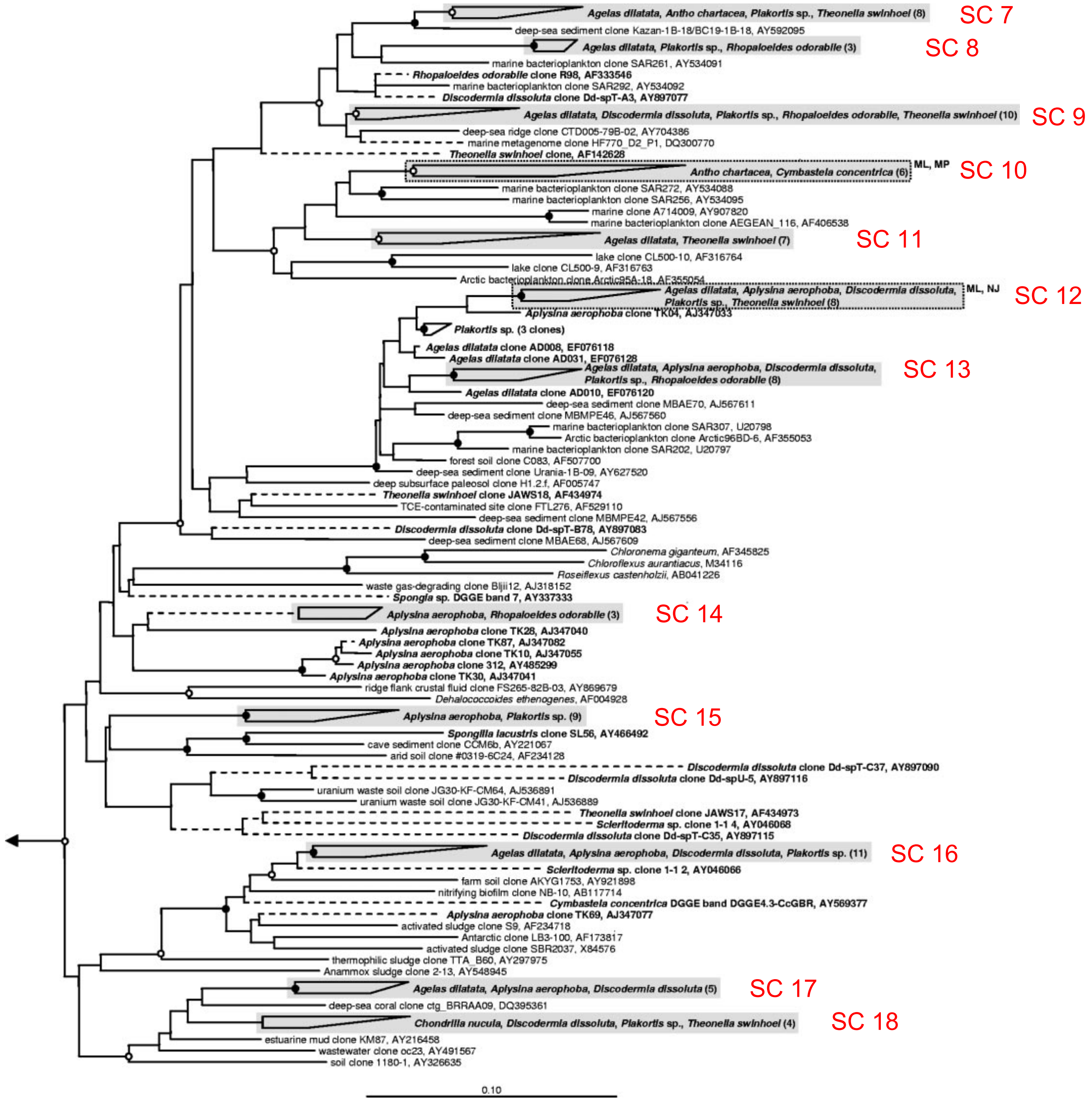

Sponge-specific clusters within the *Chloroflexi*, based on Fig. 6 from Taylor *et al.* (2007). Reproduced with kind permission of the American Society for Microbiology.

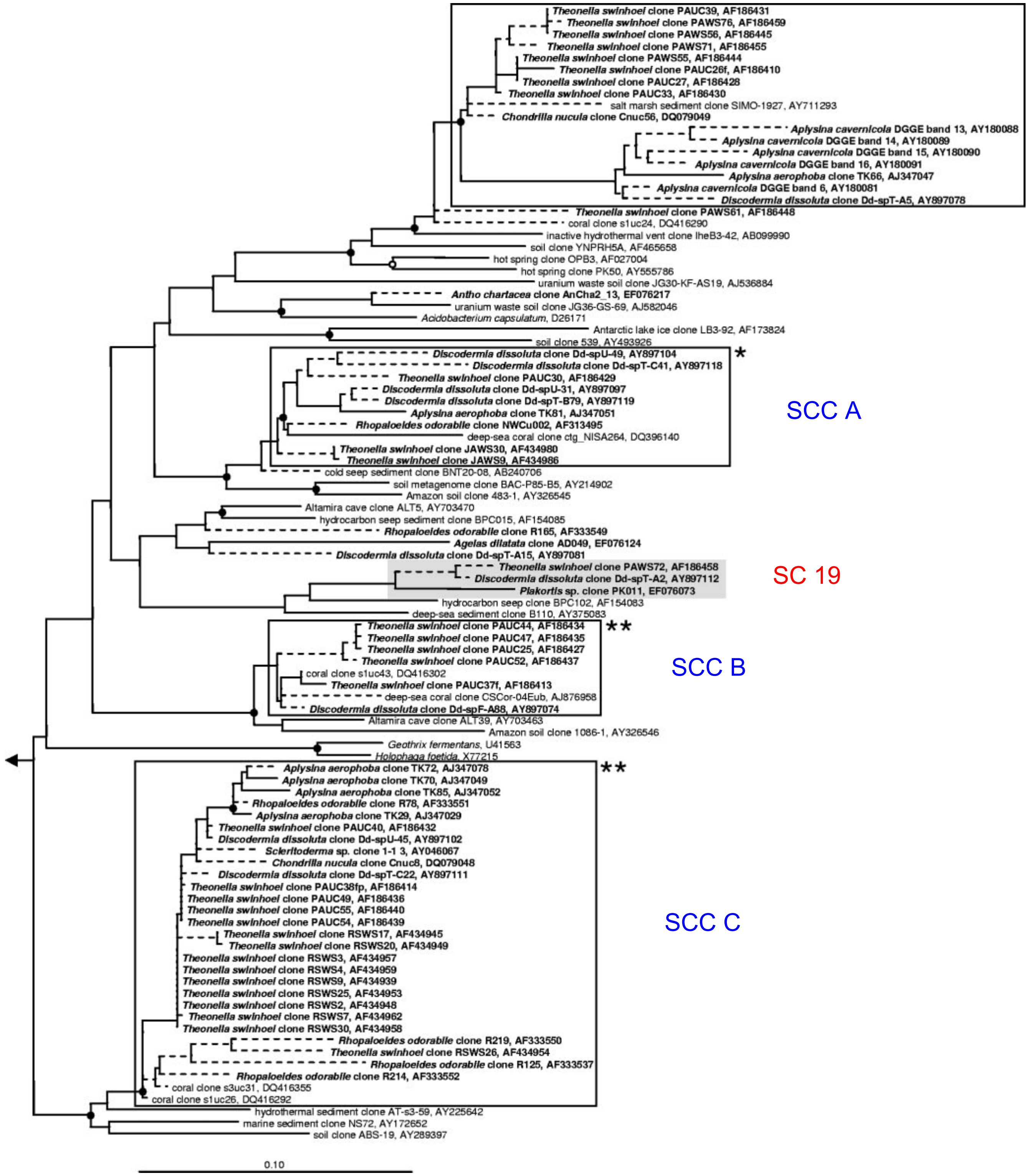

Sponge-specific clusters within the *Acidobacteria*, based on Fig. 7 from Taylor *et al.* (2007). Reproduced with kind permission of the American Society for Microbiology.

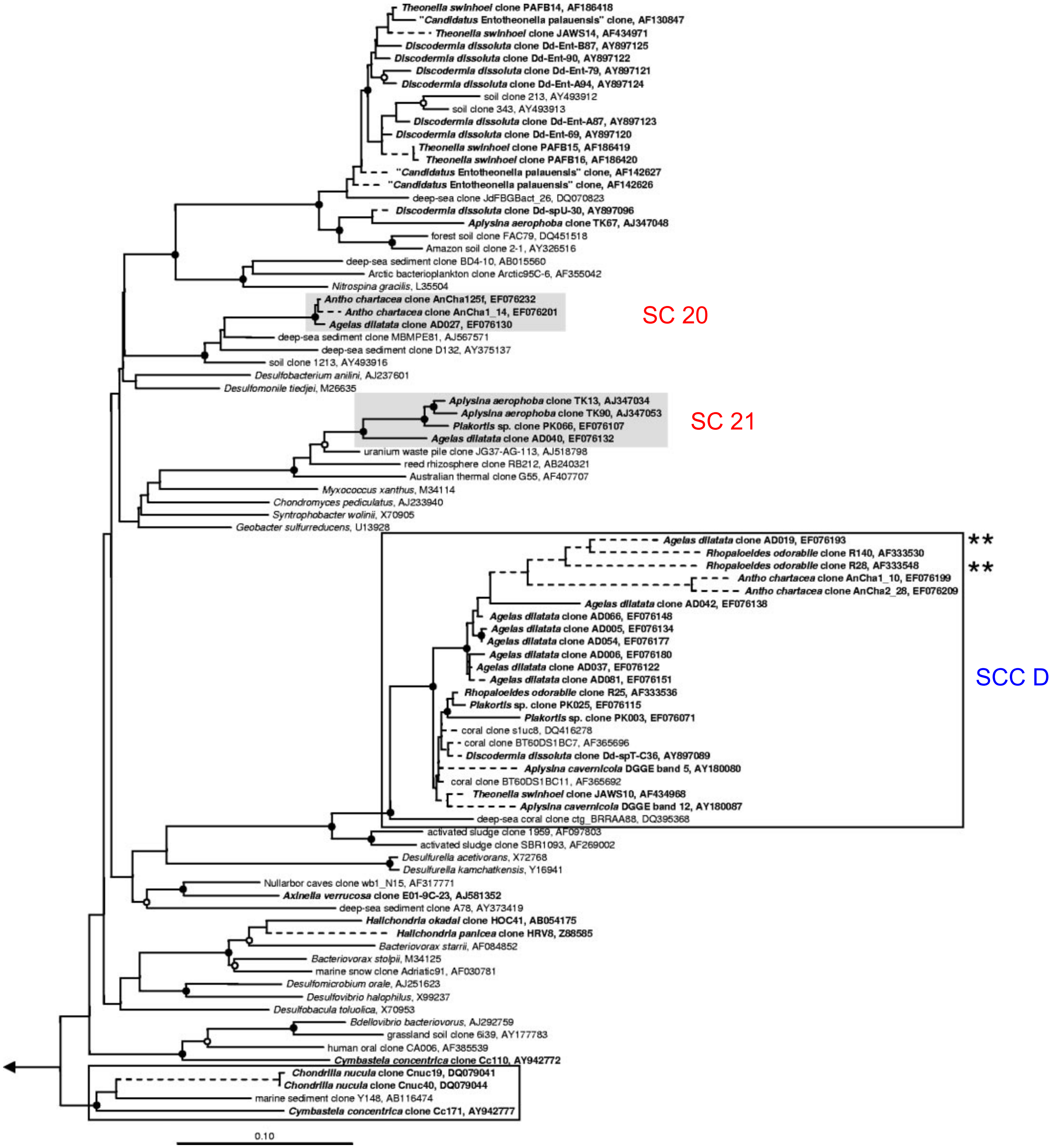

Sponge-specific clusters within the *Deltaproteobacteria*, based on Fig. 8 from Taylor *et al.* (2007). Reproduced with kind permission of the American Society for Microbiology.

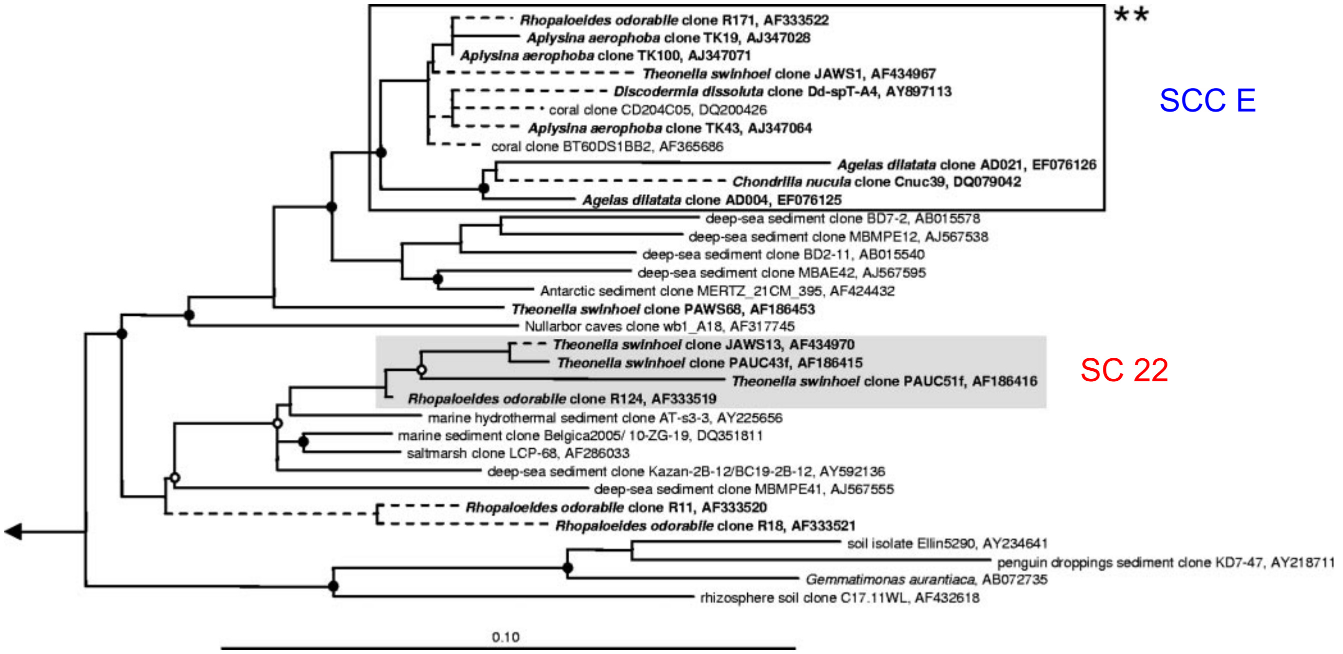

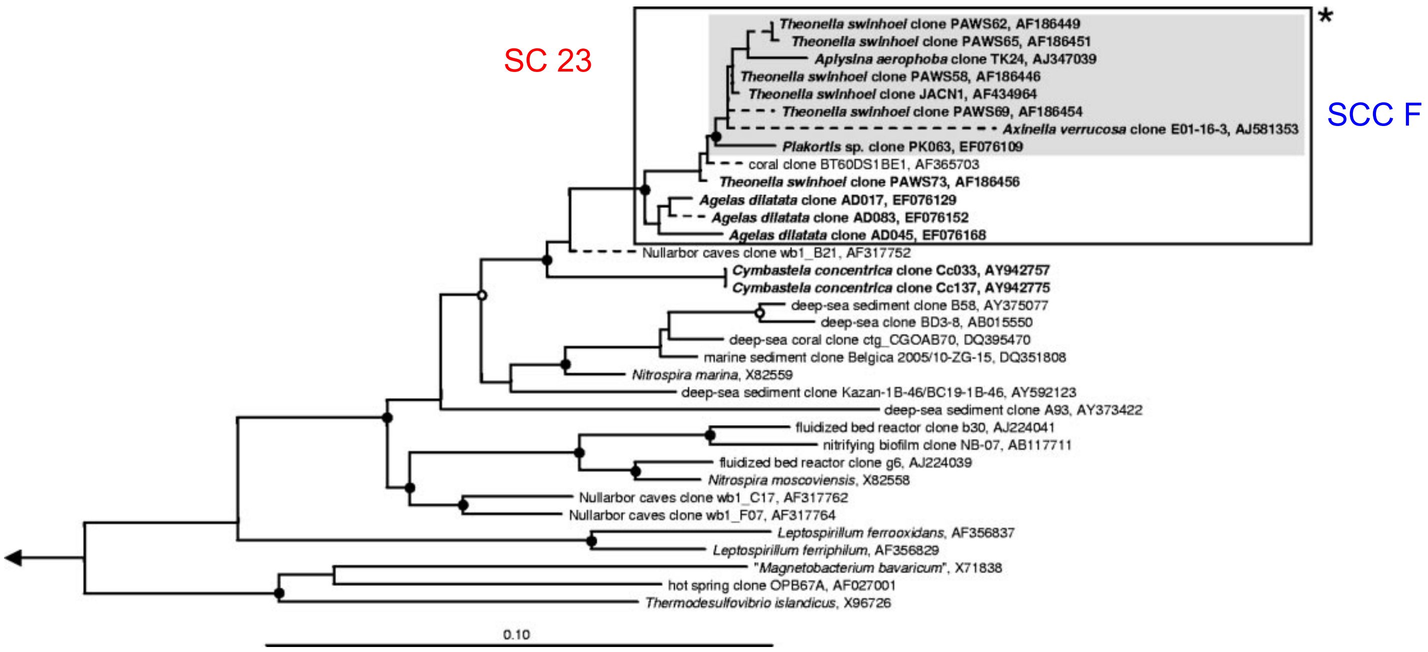

Sponge-specific clusters within the *Gemmatimonadetes* (top) and *Nitrospira* (bottom), based on Figs. 9 and 10, respectively, from Taylor *et al.* (2007). Reproduced with kind permission of the American Society for Microbiology.

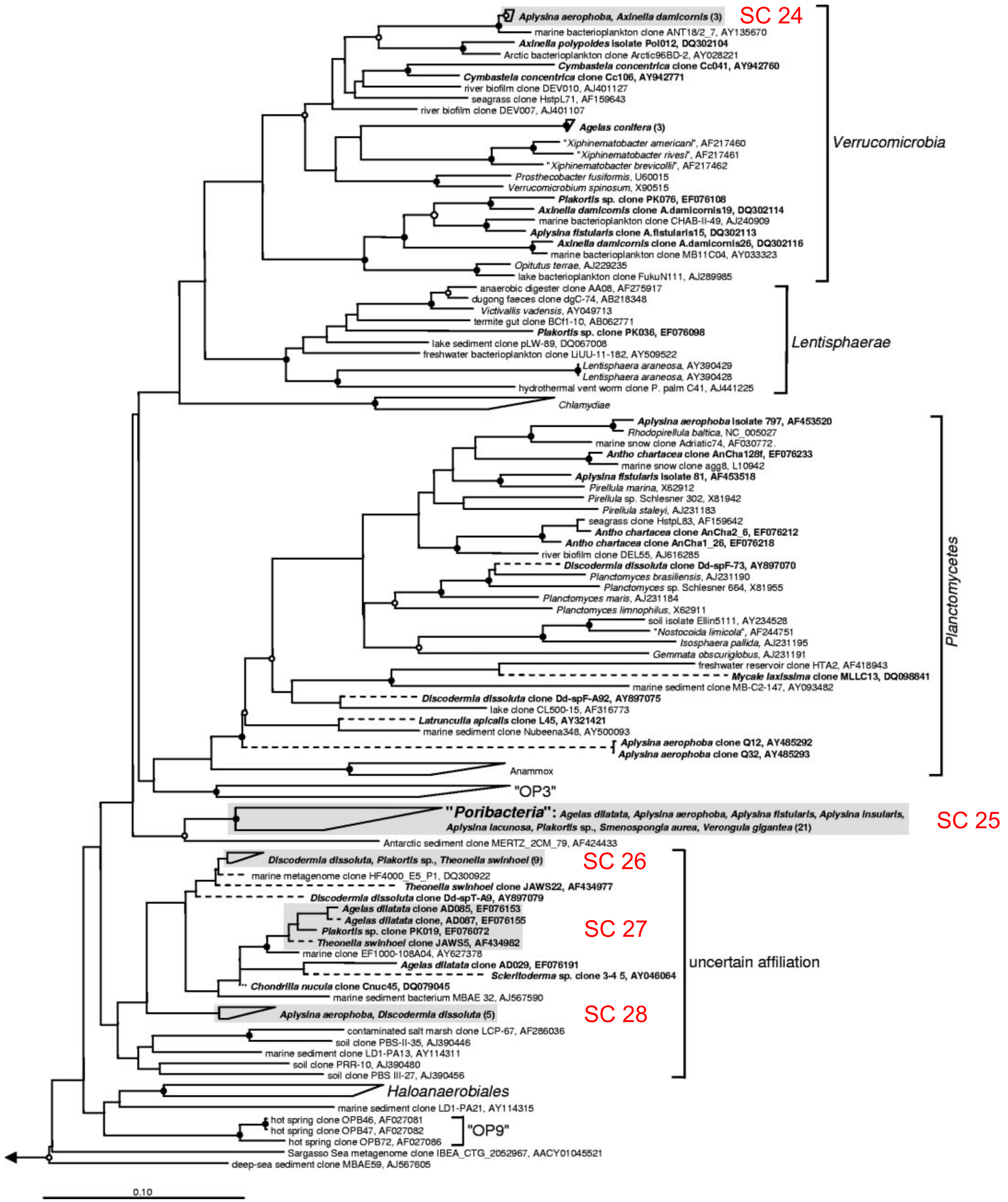

Sponge-specific clusters within the *Verrucomicrobia*, "*Poribacteria*" and a lineage of uncertain affiliation, based on Fig. 11 from Taylor *et al.* (2007). Reproduced with kind permission of the American Society for Microbiology.

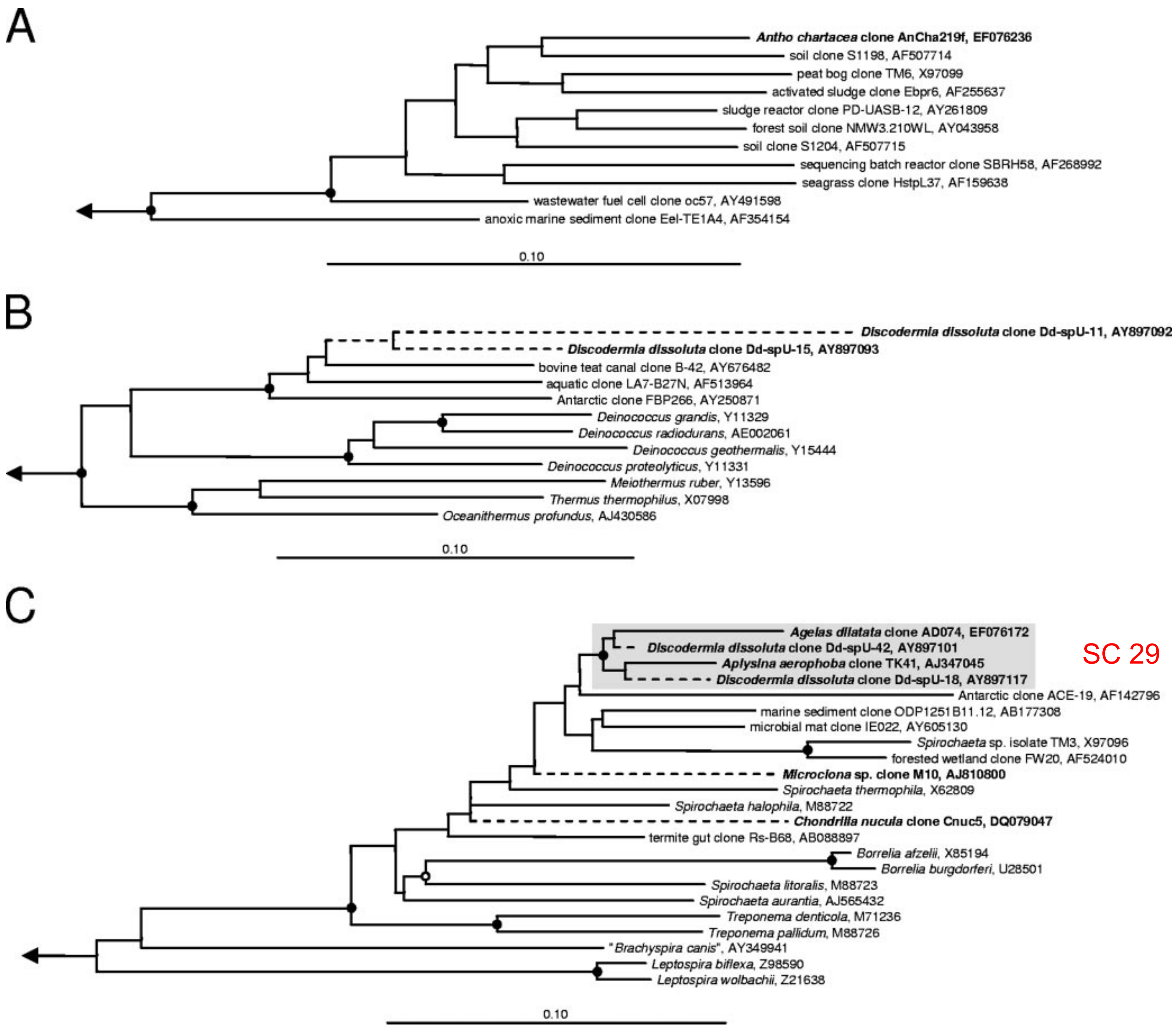

Sponge-specific clusters within the *Spirochaetes*, based on Fig. 12 from Taylor *et al.* (2007). Reproduced with kind permission of the American Society for Microbiology.

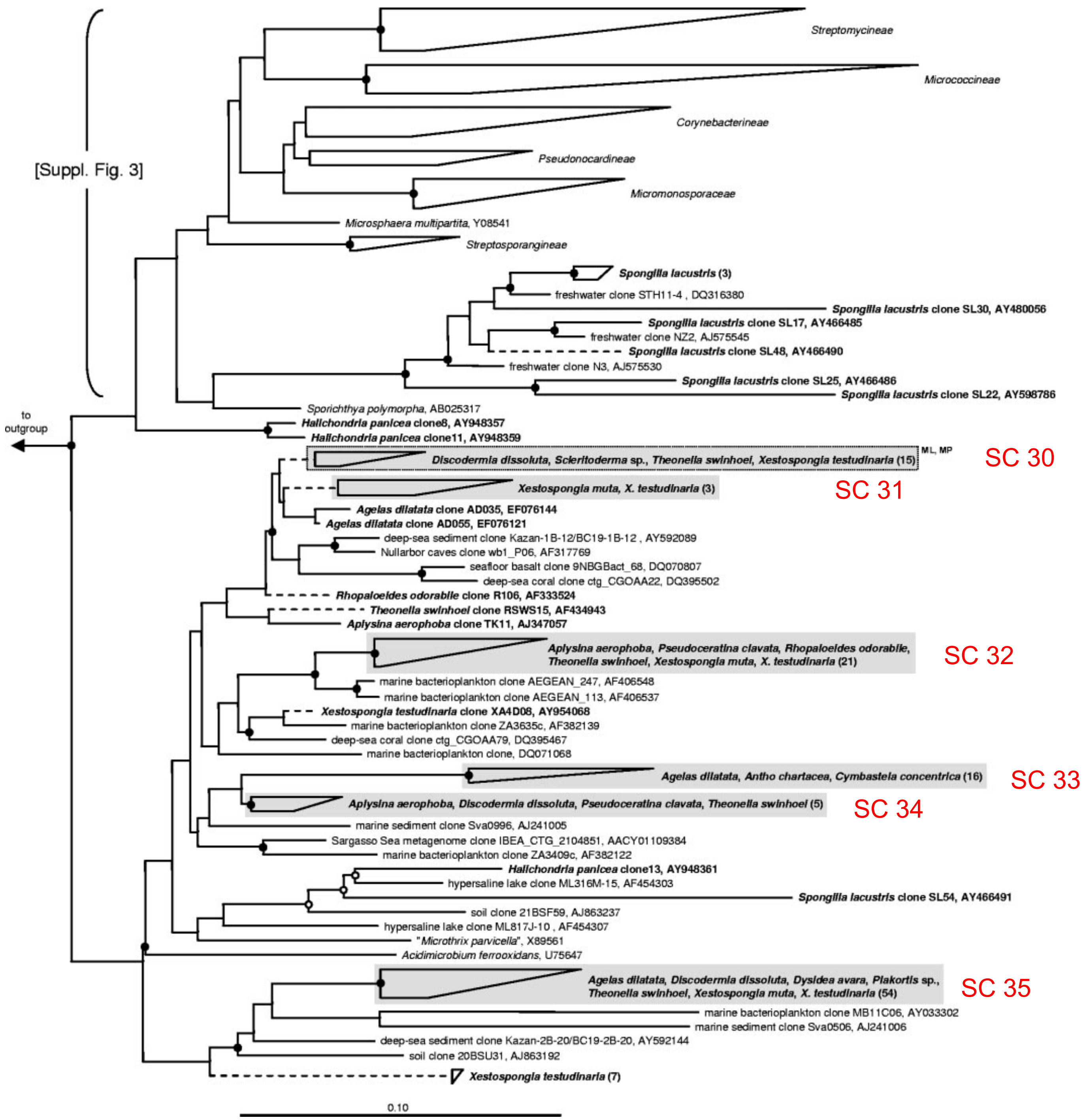

Sponge-specific clusters within the *Actinobacteria* (family *Acidimicrobiaceae*), based on Fig. 13 from Taylor *et al.* (2007). Reproduced with kind permission of the American Society for Microbiology.

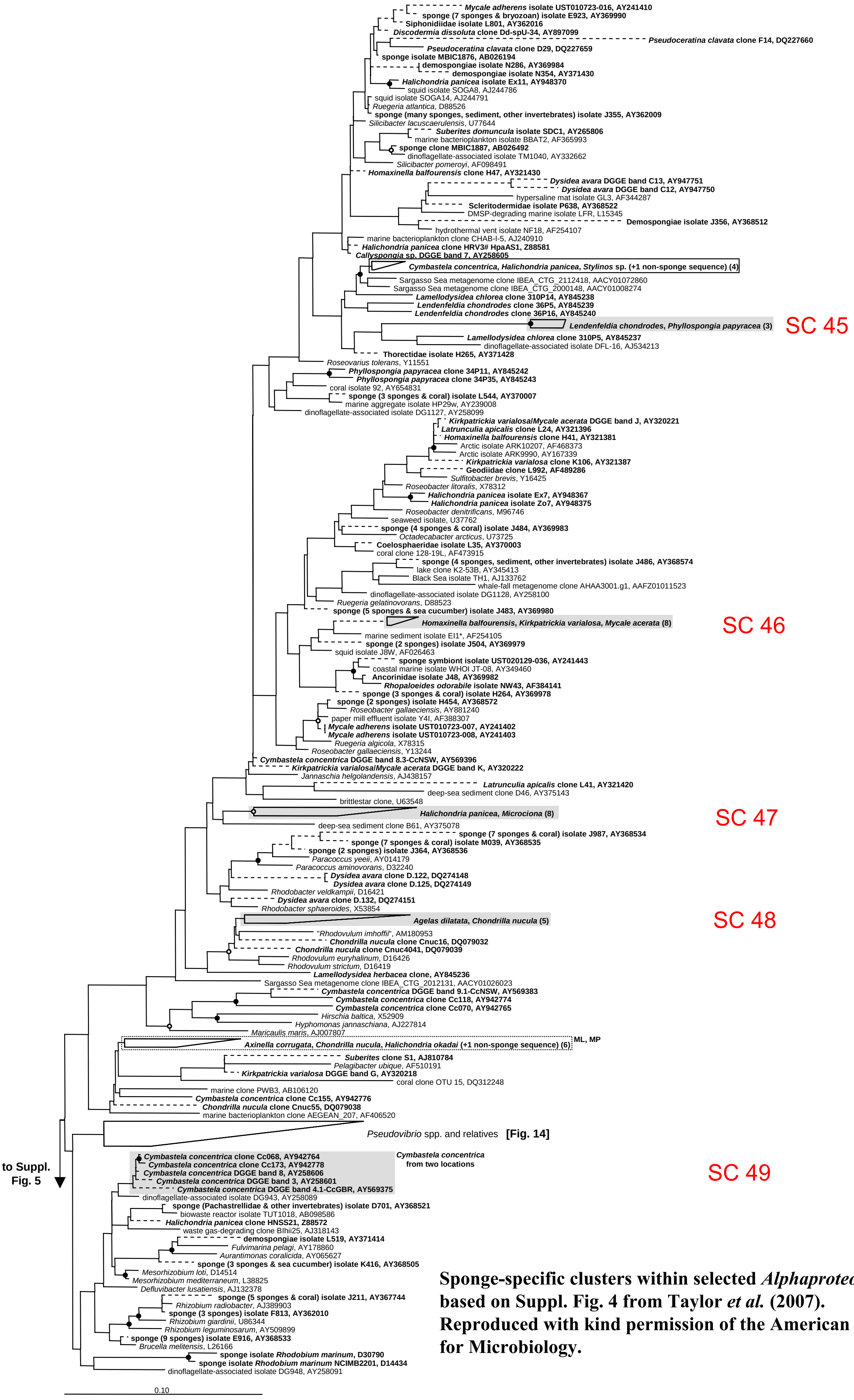

Sponge-specific clusters within selected *Alphaproteobacteria*, based on Suppl. Fig. 4 from Taylor *et al.* (2007). Reproduced with kind permission of the American Society for Microbiology.

Sponge-specific clusters within selected *Alphaproteobacteria*, based on Suppl. Fig. 5 from Taylor *et al.* (2007). Reproduced with kind permission of the American Society for Microbiology.

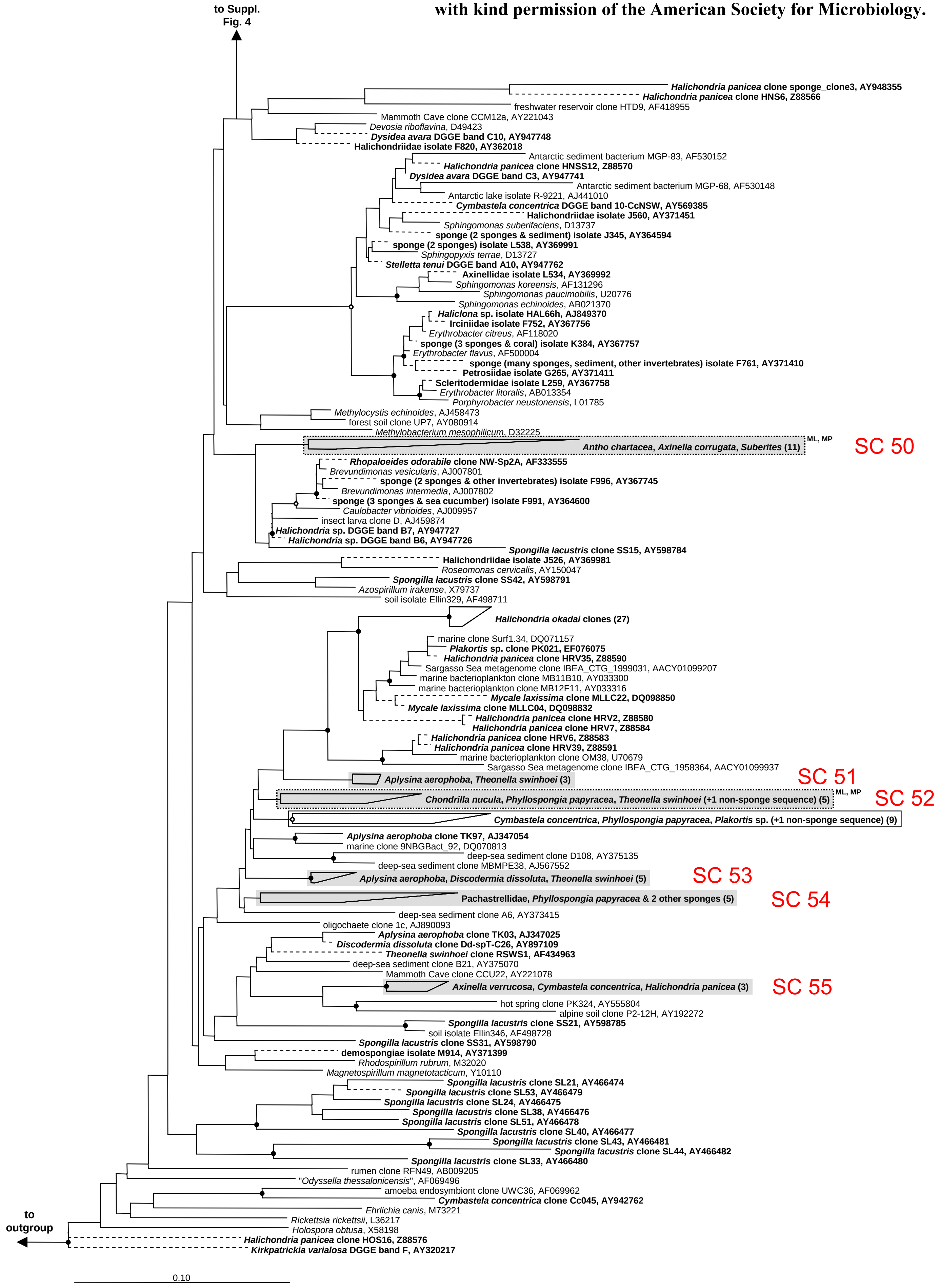

Sponge-specific clusters within selected *Gammaproteobacteria*, based on Suppl. Fig. 6 from Taylor *et al.* (2007). Reproduced with kind permission of the American Society for Microbiology.

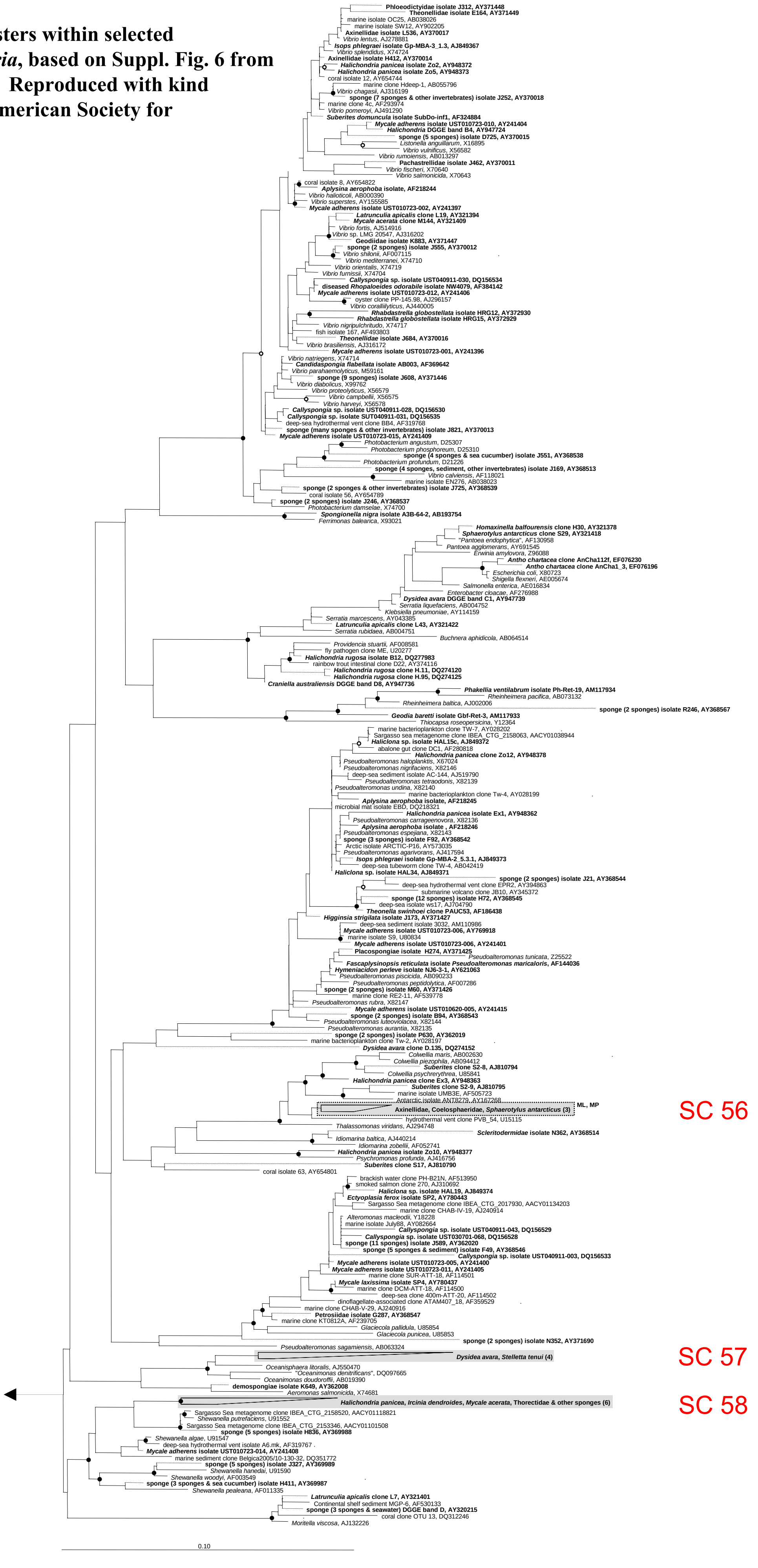

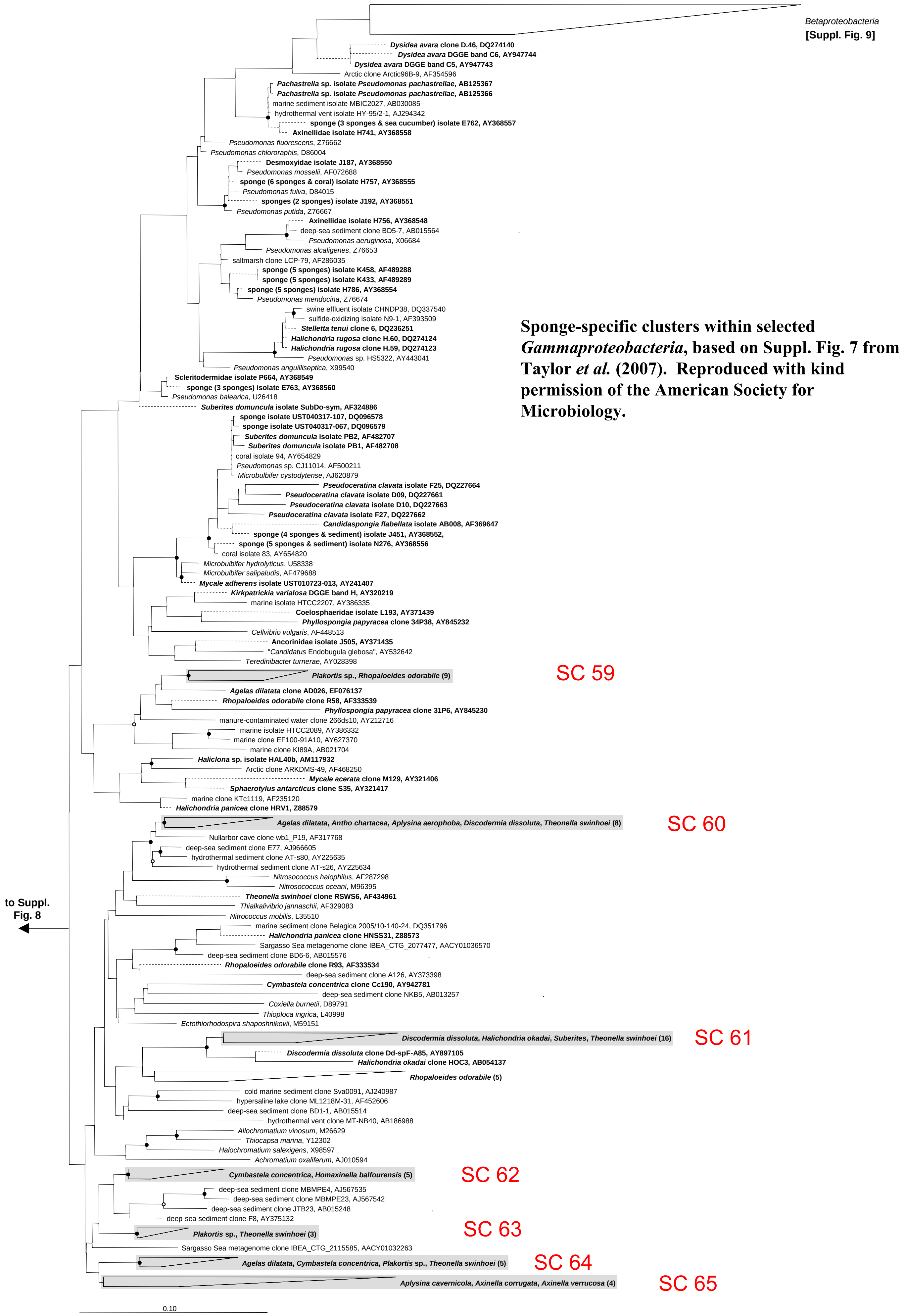

Sponge-specific clusters within selected *Gammaproteobacteria*, based on Suppl. Fig. 7 from Taylor *et al.* (2007). Reproduced with kind permission of the American Society for Microbiology.

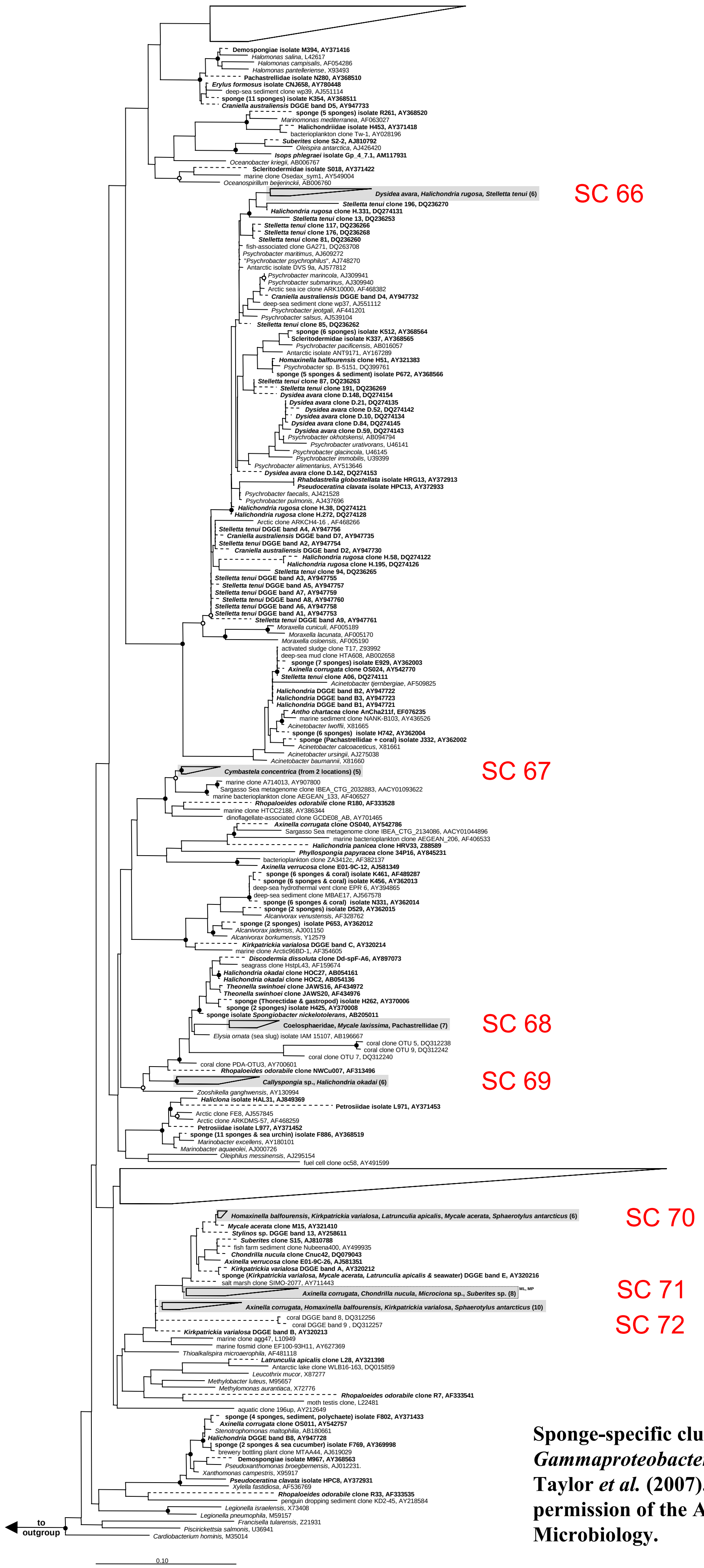

Sponge-specific clusters within selected *Gammaproteobacteria*, based on Suppl. Fig. 8 from Taylor *et al.* (2007). Reproduced with kind permission of the American Society for Microbiology.

Supporting Tables

Table S1 Taxonomic assignments of V6 sequence tags from sponge and seawater samples at the genus level. Relative abundances (in %) are denoted. Sample numbers: 1-3, *R. odorabile*; 4-6, *R. odorabile* larvae; 7-9, *I. basta*; 10-12, *I. ramosa*; 13-16, seawater.

	1	2	3	4	5	6	7	8	9	10	11	12	13	14	15	16
Achromobacter	0	0	0	0	0	0	0	0	0	0	0	0	0	0	0.02	0
Acidimicrobineae	0	<0.01	<0.01	0	<0.01	0	0	0	0	0	0	0	0	0	0	<0.01
Acidithiobacillus	0	0	0	<0.01	0	0	0	0	0	0	0	0	0	0	0	0
Acidovorax	0	0	<0.01	0.45	0.14	0.23	0	0.01	0.01	<0.01	0.01	0.03	0.46	0.05	0.07	0.02
Acinetobacter	0.03	<0.01	<0.01	0.38	0.24	1.23	<0.01	<0.01	0.02	0.01	<0.01	<0.01	<0.01	<0.01	0.24	<0.01
Actinobacillus	0	0	0	0	0	0	0	0	0	0	0	0	0	<0.01	0	0
Actinomycineae	0	0	0	<0.01	<0.01	0	0	0	0	0	0	<0.01	0	0	0	<0.01
Aeromonas	0	0	0	0	0	0.16	0	0	0	0	0	0	0	0	0	0
Aestuariibacter	0	0	0	0.17	0	<0.01	0	<0.01	0	0	0	0	0	<0.01	<0.01	<0.01
Afipia	0	0	0	<0.01	0	0	0	0	0	0.01	0	0	<0.01	0	0	0
Agarivorans	0	0	0	<0.01	0	0	0	0	0	0	0	0	0	0	0	<0.01
Agrobacterium	0	0	0	0	0	0	0	0	0	0	0	0	0	0	<0.01	0
Alcanivorax	0	0	0	<0.01	0.17	0.24	0	0	0	0	0	0	0	<0.01	0.02	0
Algicola	0	0	0	<0.01	0	0	0	0	0	0	0	0	0	0	0	0
Alistipes	0	0	0	0	0	0	0	0	0	0	0	0	0	0	0	<0.01
Alkalilimnicola	0	<0.01	0	0	0.01	<0.01	0	0	0	0	0	0	0.04	0.01	<0.01	0.04
Alkanindiges	0	0	0	<0.01	0	<0.01	0	0	0	0	0	0	0	0	<0.01	0
Alteromonas	0	0	0	0.57	0.06	0.83	0	<0.01	<0.01	0	0	0	0.16	0.04	<0.01	0.06
Amaricoccus	0	0	0	0	0	<0.01	0	0	0	0	0	0	0	0	0	0
Aminobacterium	0	0	0	0	0	0	0	0	0	0	0	0	0	0	0	<0.01
Anaerococcus	0	0	0	0	0	0	0	0	0	0	0	0	0	0	0.01	0
Ancalomicrobium	0	0	0	0.02	0	0	0	0	0	0	0	0	0	0	0	0
Aquabacterium	0	0	0.02	<0.01	0.03	0.06	0.02	0.02	<0.01	0.01	<0.01	<0.01	0.01	<0.01	0.05	0
Aquitalea	0	0	0	0	0	0	0	<0.01	<0.01	0	0	0	0	0	0	0

**Arcobacter**	0	0	0	0	0	0	0	0	0	0	0	0	<0.01	<0.01	0	0
**Arsenophonus**	0	0	0	0	0	0	0	0	0	0	0	0	<0.01	0	0	0
**Aurantimonas**	0	0	0	0	0	0	0	0	0	0	0	0	0	<0.01	0	0
**Azonexus**	0	0	0	<0.01	0	<0.01	0	0	0	0	0	0	0	0	0	0
**Azospira**	0	0	0	0	0	0	0	<0.01	0	0	0	0	0	0	0	0
**Bacteriovorax**	0.01	0	0	0	0	<0.01	0	0	<0.01	0	0	0	0.02	0.02	0.02	<0.01
**Bacteroides**	0	0	0	<0.01	0	0	0	0	0	0	0	0	0	0	0	0
**Balneola**	0	0	0	<0.01	0	0.02	0	0	0	0	0	0	0	0	0	0
**Bartonella**	0	0	0	0	0	0	0	<0.01	0	0	0	0	0	0	0	<0.01
**Bdellovibrio**	0	0	0.19	<0.01	0	0	<0.01	0	0	0	0	0	0	<0.01	0	0
**Belliella**	0	0	0	0	0	0	0	0	0	0	0	0	0	0	<0.01	0
**Bifidobacteriaceae**	0	0	0	0	0	0	0	0	0	0	0	0	<0.01	0	<0.01	0
**Blastopirellula**	0	0	0	<0.01	0	0.02	<0.01	0	0	0	0	<0.01	0.05	0.04	0.04	0.02
**Bosea**	0	0	0	0.01	0	0	0	0	0	0	0	0	0	0	0	0
**Bradyrhizobium**	0	0	0	0.02	<0.01	<0.01	0	0.01	0	0.01	<0.01	0	0	<0.01	0.08	0
**Brenneria**	0	0	0	0	0	0	0	0	0	0	0	0	0	<0.01	0	0
**Brevundimonas**	0.01	0	0	0.02	0.01	<0.01	<0.01	0.04	0	0.05	0.01	<0.01	0.07	<0.01	0	0
**Brumimicrobium**	0	0	0	0	0	<0.01	0	0	0	0	0	0	0	<0.01	0	0
**Bulleidia**	0	0	0	0	0	<0.01	0	0	0	0	0	0	0.08	<0.01	0	0
**Burkholderia**	0	0	0	0	0	0	0	0	0	<0.01	0	<0.01	0	0	<0.01	0
**Buttiauxella**	0	0	0	<0.01	0	<0.01	0	0	0	0	0	0	0	0	0	0
**Caedibacter**	0	0	0	0	0.02	<0.01	0	0	0	0	0	0	0.04	<0.01	0	<0.01
**Caldilineacea**	2.05	1.98	1.76	0.92	1.92	0.17	0	0.11	0	2.18	3.61	2.92	0.03	0.03	0.02	0.04
**Caldimonas**	0	0	0	0	<0.01	0	0	<0.01	0	0	0	0	0	0	0	0
**Caldithrix**	0	0	0	0	0	0	0	0	0	0	0	0	0	<0.01	0	<0.01
**Cardinium**	0	0	0	0	0	<0.01	0	0	0	0	0	0	0	<0.01	0	0
**Cardiobacterium**	0	0	0	<0.01	0	0	0	<0.01	0	0	0	0	0	0	0	<0.01
**Caulobacter**	0	0	0	<0.01	0	0	0	0	0	0	0	0	<0.01	0	0	0
**Cellulophaga**	0	0	0	0	0	<0.01	0	0	0	0	0	0	0	0	0	0
**Cellvibrio**	0	0	0	0.03	0.02	0.05	0	0	0	0	0	0	0	<0.01	0	0
**Cetobacterium**	0	0	0	0	0	0	0	0	0	0	0	0	<0.01	<0.01	0.01	0

**Chloroplasts**	0.06	0.02	0.08	22.1	5.61	25.6	0.62	0.34	0.54	0.02	0.03	0.03	20.1	14.4	19.8	11.5
**Chromobacterium**	0	0	0	<0.01	0	0	0	0	0	0	0	0	0	0	0	0
**Chromohalobacter**	0	<0.01	0	0.02	0.01	0.06	0	0	0	0	<0.01	0	0	0	0	0
**Chryseobacterium**	0	0	0	0.04	<0.01	0.05	0	0	0	0	0	0	4.91	0.28	0.04	0.03
**Citrobacter**	0	0	0	0	0	<0.01	0	0	0	0	0	0	0	<0.01	<0.01	<0.01
**Cloacibacterium**	0.06	0.01	0.06	0.22	0.11	0.33	0.01	0	0.03	0.02	0.03	0.03	0.03	<0.01	0.07	0
**Colwellia**	0	0	0	0.39	<0.01	0.63	<0.01	0	0	0	0	0	0.05	0.02	0.03	0.03
**Comamonas**	0	0	0	0.03	0	0	0	0	0	0	0.01	0	0	0	0	<0.01
**Corynebacterineae**	0	0.03	0.01	0.25	0.06	0.31	0	0	0	<0.01	0	0	0.09	<0.01	0.18	0
**Coxiella**	0	<0.01	0	0	0.01	0.02	<0.01	<0.01	0	0	0	0	0.06	0.07	0.02	0.05
**Cryomorpha**	0	0	0	0	0	<0.01	0	0	0	0	<0.01	0	0	<0.01	<0.01	0
**Cupriavidus**	0	0	<0.01	0	0.01	0	<0.01	<0.01	0.01	0	0.01	0	<0.01	0	0.05	0
**Curvibacter**	0	0	0.02	0	0.02	0	<0.01	<0.01	0.01	0.02	0	<0.01	0	0	0	0
**Cycloclasticus**	0	<0.01	0	0	0	0	0	0	0	0	0	0	0	0	0	0
**Cystobacteraceae**	0.84	0.49	0.59	0.13	0.29	0.01	0	0	0	0.05	0.09	0	0	0	0	0
**Cytophaga**	0	0	0	0	0	<0.01	0	0	0	0	0	0	0	0	0	0
**Dechloromonas**	0	0	0	0.09	0	0.01	0.01	0.03	0	0	0	0	0	0	0	0
**Delftia**	0	0	0	<0.01	0.02	0.06	<0.01	0	0	0	<0.01	0	<0.01	0	0.03	0
**Desulfarculus**	0	0	0	0	0	0	0	0	0	0	0	0	0	0	0	<0.01
**Desulfobacter**	0	0	0	0	0	0	0	0	0	0	0	0	0	<0.01	<0.01	0
**Desulfobacterium**	0	0	0	0	0	<0.01	0	0	0	0	0	0	0	<0.01	0	<0.01
**Desulfobacula**	0	0	0	0	0	0	0	0	0	0	0	0	0	<0.01	0	0
**Desulfobulbus**	0	0	0	0	0	0	0	0	0	0	0	0	0	0	0	<0.01
**Desulfocapsa**	0	0	0	0	<0.01	0	0	0	0	0	0	0	0	0	0	<0.01
**Desulforhopalus**	0	0	0	<0.01	0	<0.01	0	0	0	0	0	0	<0.01	<0.01	0	<0.01
**Desulfosarcina**	0	0	0	0	0	0	0	0	0	0	0	0	0	<0.01	0	<0.01
**Desulfovibrio**	0	0	0	0	0	0	0	0	0	0	0	0	<0.01	<0.01	0.02	<0.01
**Desulfuromonas**	0	0	0	<0.01	0	0	0	0	0	0	0	0	0	0	0	0
**Devosia**	0	0	0	0	0	<0.01	0	0	0	0	0	<0.01	0	<0.01	0	<0.01
**Diaphorobacter**	0.31	0.04	0.1	0.3	0.09	0.07	0.03	0.05	0.13	0.03	0.03	0.04	<0.01	0.01	0.32	<0.01
**Dyadobacter**	0	0	0	0	0	<0.01	0	0	0	0	0	0	0	0	0	0

**Elizabethkingia**	0	0	0	<0.01	0.03	<0.01	0	0	0	0	0	0	0	0	<0.01	0
**Emticicia**	0	0	0	0	0	<0.01	0	0	0	0	0	0	0	0	0	0
**Enhydrobacter**	0	0	0	<0.01	0	0	0	0	0	0	0	0	0	0	0	0
**Ensifer**	0	0	0	0	0	0	0	0	0	0	0	0	0	<0.01	0	0
**Enterobacter**	0	0	0	<0.01	0	<0.01	0	0	0	0	0	0	0.1	0.02	<0.01	0
**Enterococcus**	0	<0.01	0	0	0	0	0	0	0	0	0	0	0	0	0.02	0
**Enterovibrio**	0	0	0	<0.01	0.01	0	0	0	0	0	0	0	0.01	<0.01	0	0.01
**Erwinia**	0	0	0	0	0	0.03	0	0	0	<0.01	0	0	0	<0.01	<0.01	0
**Erythrobacter**	0	0	0	0.06	0.03	0.96	<0.01	0	0	0	0	0	0.01	0.01	0.03	0.01
**Exiguobacterium**	0	0	0	<0.01	<0.01	0.67	0	0	0	0	0	<0.01	0	<0.01	0.18	<0.01
**Fabibacter**	0	0	0	0	0	0	0	0	0	0	0	0	0	<0.01	0	0
**Faecalibacterium**	0	0	0	0	0	0	<0.01	0	0	0	0	0	0	0	0	0
**Ferrimonas**	0	0	0	<0.01	0	0.02	0	0	0	0	0	0	0.03	<0.01	<0.01	0.01
**Filomicrobium**	0	<0.01	0	0	0	<0.01	0	<0.01	0	0	0	0	0.01	0	0	<0.01
**Flammeovirga**	0	0	0	0.02	<0.01	0.08	0	0	0	0	0	0	0.07	0.04	0.04	0.03
**Flavimonas**	0	0	0	0	0	0	0	0	0	0	0	0	0	0	0	<0.01
**Flavobacterium**	0.01	0	0	0.03	0.05	0.02	0	<0.01	0	0	0.01	<0.01	0	<0.01	0	0
**Flectobacillus**	0	0	0	0.02	0.03	0.12	0	0	0	0	0	0	0	0	0	0
**Flexibacter**	0	0	0	0	0	0	0	0	0	0	0	0	0	<0.01	0	0
**Fluviicola**	0	0	0	0.03	<0.01	0.05	0	0	0	0	0	0	0.03	0.06	0.01	0.02
**Francisella**	0	0	0	0.07	0.01	0.07	0	<0.01	0	0	0	0	0.02	<0.01	<0.01	0.01
**Frankineae**	0	0	0	0.04	0.02	<0.01	0	0	0	0	0	0	0.02	0	0.01	0
**Fusibacter**	0	0	0	0	0	0	0	0	0	0	0	0	0	<0.01	0	0
**Fusobacterium**	0	0	0	0	0	0	0	0	0	0	0	0	0	<0.01	0	0
**Gemmatimonas**	0.06	0	0	0	0	0	0	0	0	0	0	0	0	<0.01	0	<0.01
**Gillisia**	0	0	0	<0.01	0	<0.01	0	0	0	0	0	0	0	<0.01	0	0
**Glaciecola**	0	0	0	0	0	0.03	0	0	0	0	0	0	<0.01	0	0	0
**Gp10**	0	0	0	<0.01	<0.01	0.03	0	<0.01	0	0	0	0	<0.01	<0.01	0	0.03
**Gp11**	2	1.86	1.52	0.47	0.38	0.11	0.01	0.12	0	0.53	0.2	0.17	0	0	0	0
**Gp17**	0	0	0	0	0	0	0	0	0	0	0	0	0	<0.01	0	0
**Gp21**	0.58	0.97	0.56	0.09	0.14	0.06	0	0.03	0	0.12	0.14	0	0	<0.01	0	0

**Gp22**	0	0	0	0	<0.01	0.03	0	0	0	0	0	0	0.03	0.01	0	0.04
**Gp23**	0	0	0	0	0	0	0	0	0	0	0	0	<0.01	<0.01	0	<0.01
**Gp26**	0	0	0	0	0	0	0	0	0	0	0	0	0	<0.01	0	<0.01
**Gp3**	0.04	0.01	0.01	0	0	<0.01	0	0.01	0	0	0	0	0.02	0	0.03	<0.01
**Gp4**	0	0	0	0	0	0	0	0	0	0	0	0	0	<0.01	0	0
**Gp6**	3.6	4.63	6.06	1.15	2.79	0.23	<0.01	0.04	0.03	1.57	0.59	0.5	0	0	0.01	<0.01
**Gp9**	0	0	0	0	0	0	0	0	0	0	0	0	<0.01	<0.01	0	0.02
**Gramella**	0	0	0	0	0	<0.01	0	0	0	0	0	0	0	0	<0.01	0
**Haemophilus**	0	0	0	0	0	<0.01	0	0	0	0	0	0	0	<0.01	0	0
**Haliangiaceae**	0	0	0	0	<0.01	0	0	0	0	0	0	0	0	0	0	0
**Haliscomenobacter**	0	0	0	<0.01	0	0.02	0	0	0	0	0	0	0	<0.01	<0.01	<0.01
**Halomonas**	0	0	0	0.07	0.03	0.64	0	0	0	0	0	0	<0.01	<0.01	0	<0.01
**Halothiobacillus**	0	<0.01	0	<0.01	0	<0.01	0	0.02	0	0	0	0	<0.01	<0.01	<0.01	<0.01
**Herbaspirillum**	0	0	0	<0.01	<0.01	0	0	0	0	0	0	0	0	0	0	0
**Herpetosiphon**	0	0	0	0	0	<0.01	0	0	0	0	0	0	0	0	0	0
**Hoeflea**	0	<0.01	0	0	0	0	0	0.02	0	0	1.11	0.33	<0.01	0	0	0
**Hongiella**	0	0	0	0	0.01	0.02	0	0	0	0	0	0	0	<0.01	0	0
**Hydrocarboniphaga**	0	0	0	0	0	<0.01	0	0	0	0	0	0	0	0	0	0
**Hydrogenophaga**	0	0	0	0	0	0	0	0	0	0	0	0	0	0	0.02	0
**Hydrogenophilus**	0	0	0	0	0	<0.01	0	0	0	0	0	0	0	0	0	0
**Hymenobacter**	0	0	0	0	0	0	0	0	0	0	0	0	0	<0.01	<0.01	0
**Hyphomicrobium**	0	0	0	0	0	0	0	0	0	0	0	0	0	0	0	<0.01
**Hyphomonas**	0	0	0	<0.01	0	0.07	0	0	0	0	0	0	<0.01	0	0	0
**Idiomarina**	0	0	0	0.08	0.04	0.43	0	0	0	0	0	0	0.04	<0.01	0.02	0.02
**Ilyobacter**	0	0	0	0	0	<0.01	0	0	0	0	0	0	0	0	0	0
**Incertae sedis 10**	0	0	0	<0.01	0	0	0	0	0	0	0	0	0	0	0	0
**Inquilinus**	0	0	0	0	<0.01	0	0	0	0	0	0	0	<0.01	<0.01	0	0
**Isosphaera**	0	0	0	0	0	<0.01	0	0	0	0	0	0	0	0	0	0
**Janthinobacterium**	0	0	0	0	0	0	0	0	0	<0.01	0	0	<0.01	<0.01	0.02	0
**Jeotgalibacillus**	0	0	0	0	0	0.02	0	0	0	0	0	0	0	0	0	0
**Jeotgalicoccus**	0	0	0	<0.01	0	0	0	0	0	0	0	0	0	0	0	0

**Kaistia**	0	0	0	0	0	<0.01	0	0	0	0	0	0	0	0	0	0
**Klebsiella**	0	0	0	<0.01	0	0.01	0	0	0	0	0	0	0	0	0	0
**Kordia**	0	0	0	0	0	0	0	0	0	0	0	0	0	0	0	<0.01
**Kordiimonas**	0	0	0	<0.01	0	0.02	0	0	0	0	0	0	<0.01	<0.01	0	0.02
**Labrys**	0	0	0	0	0	0	0	0	0	0	0	0	0	<0.01	0	0
**Laceyella**	0	0	0	0	0	0	<0.01	0	0	0	0	0	0	0	0	0
**Lactobacillus**	0	0	0	0	0	0	0	0	0	0	0	0	0	<0.01	0.03	0
**Lactococcus**	0	0	0	0	0.02	0	0	0	0	0	0	0	0	0	0	0
**Legionella**	0	0	0	0	0	<0.01	0	0	<0.01	0	0	0	0	0	<0.01	0
**Leptospira**	0	0	0	0	0	0	0	0	0	0	0	0	<0.01	0	0	0
**Leptothrix**	0	0	0	0.17	0.15	0.34	0	0	0	0	0	0	0	0	0	0
**Lewinella**	0	0	0	<0.01	0.01	0.03	0	0	0	0	0	0	<0.01	<0.01	0.02	0.02
**Limnobacter**	0	0	0	<0.01	0	0	0	0	0	0	0	0	0	0	0	0
**Loktanella**	0	0	0	0.03	0.01	0.06	0	0	0	0	0	0	0	<0.01	0	<0.01
**Lutibacter**	0	0	0	0	0	<0.01	0	0	0	0	0	0	0.05	<0.01	<0.01	0
**Magnetobacterium**	0	0	0	0	0	0	0	0	0	0	0	0	0	<0.01	0	0
**Malikia**	0	0	0	0	0	0	0	0	0	0	0	0	0	0	0.03	0
**Mannheimia**	0	0	0	<0.01	0	0.04	0	0	0	0	0	0	0	<0.01	0.02	<0.01
**Maricaulis**	0	<0.01	0	0	0	0.01	0	0	0	0	0	0	0	<0.01	0	<0.01
**Marinicola**	0	<0.01	0	0	0	0	0	0	0	0	0	0	<0.01	<0.01	0	<0.01
**Marinimicrobium**	0	0	0	0	<0.01	0.04	0	0	0	0	0	0	0	<0.01	0	0
**Marinobacter**	0	0	0	0.07	0.06	0.63	0.02	0.05	0.01	0	0	0	<0.01	<0.01	0.1	0
**Marinomonas**	0	0	0	0.16	0	0.04	0	0	0	0	0	0	<0.01	<0.01	0	0
**Massilia**	0	0	0	0.02	0.02	0.02	0	0	0	0	0	0	<0.01	0	0.02	0
**Megamonas**	0	0	0	0	0	0	0	0	0	0	0	0	0	0	0.04	0
**Mesorhizobium**	0.86	0.47	0	0.45	0.91	<0.01	0	<0.01	0	0.52	0	<0.01	<0.01	<0.01	0.02	<0.01
**Methylobacter**	0	0	0	<0.01	0	<0.01	0	0	0	0	0	0	0	<0.01	0	0
**Methylobacterium**	0	0	0	<0.01	<0.01	0	0	<0.01	<0.01	0	0	0	0.05	<0.01	0.01	0.02
**Methylomonas**	0	0	0	0	0	<0.01	0	0	0	0	0	0	0	0	0	0
**Methylophilus**	0	0	0	0.02	<0.01	0.01	0	<0.01	0	0	0	<0.01	<0.01	0	0	<0.01
**Microbulbifer**	0	0	0	0.05	0.03	0.22	0.02	0	0	0	0.01	0	0.13	0.06	0.12	0.08

**Micrococcineae**	0.01	0.01	<0.01	0.69	0.31	0.71	0	0	0.03	<0.01	0	0	0.22	0.02	0.23	<0.01
**Microscilla**	0	0	0	0	0	<0.01	0	0	0	0	0	0	0	0	0	0
**Mitochondria**	0	0	0	0	0	0	0	0	0	<0.01	0	0	0	0	0	0
**Moraxella**	0	0	0	<0.01	0	0	0	0	0	0	0	0	0	0	0	0
**Moritella**	0	0	0	0.02	0	0	0	0	0	0	0	0.02	0	<0.01	0	0
**Muricauda**	0	0	0	0	0	0.02	0	<0.01	0	0	0	0	0.02	<0.01	0.01	<0.01
**Mycoplana**	0	0	0	0	0	<0.01	0	0	0	0.01	0	0	<0.01	<0.01	0	0
**Mycoplasma**	0	0	0	0	0	<0.01	0	0	0	0	0	0	0.03	<0.01	<0.01	0
**Myroides**	0	0	0	<0.01	0	0	0	0	0	0	0	0	0	<0.01	<0.01	<0.01
**Nannocystaceae**	0	<0.01	0	0	<0.01	0.04	0	<0.01	0	0	0	0	0.03	0.03	0.01	0.04
**Neisseria**	0	0	0	0	0	0	0	0	0	0	0	0	0	0	0.03	0
**Neptunomonas**	0	0	0	<0.01	0	<0.01	0	0	0	0	0	0	<0.01	0.01	0	0
**Nesiotobacter**	0	0	0	0	0	0.02	<0.01	0	0	0	0	0	0	<0.01	0	<0.01
**Niastella**	0	0	0	0	0	0	0	0	0	0.01	0	0	<0.01	<0.01	<0.01	0
**Nitratireductor**	0	0	0	0	0	<0.01	0	0	0	0	0	0	<0.01	0	0	0
**Nitrincola**	0	0	0	<0.01	0	0	0	0	0	0	0	0	0	<0.01	0	0
**Nitrospina**	0	0	0	0	0	0	0	0	0	0	0	0	<0.01	<0.01	0	0
**Nitrospira**	2.37	2.94	2.26	2.09	0.66	0.17	0	0.02	<0.01	0.83	3.42	0.44	0	<0.01	0	0
**Novosphingobium**	0	0	0	0.03	0	<0.01	0	<0.01	<0.01	0	0	0	0.65	0.16	0.13	0.09
**Oceanicaulis**	0	0	0	0	0.01	<0.01	0	0	0	0	0	0	0	0	0	0
**Oceanimonas**	0	0	0	0	0	0.32	0	0	0	0	0	0	<0.01	0	0	0
**Oceanospirillum**	0	0	0	0	0	0	0	0	0	0	0	0	0.01	0	0	0
**Odyssella**	0	0	0	<0.01	0	0	0	0	0	0	0	0	0	0	0	0
**Oleispira**	0	0	0	0.13	0	0	0	0	0	0	0	0	0	0	0	0
**Opitutus**	0	0	<0.01	0.05	0.03	0.15	0.04	0.07	0.06	0.01	<0.01	0	0.74	1.07	0.62	1.01
**Orientia**	0	0	0	0	0	0	0	0	0	0	0	0	0	0	0	<0.01
**Owenweeksia**	0	0	<0.01	0	0	0.04	<0.01	<0.01	<0.01	0	0	<0.01	0.26	0.6	0.12	0.41
**Palleronia**	0	0	0	<0.01	0	0	0	0	0	0	0	0	0	<0.01	0	0
**Pantoea**	0	0	0	0	0	<0.01	0	0	0	0	0	0	0	0	0	<0.01
**Paracoccus**	0	0	0	0.49	0.01	0.05	0	0	0	0	0	0	0.02	<0.01	0.03	<0.01
**Parvibaculum**	0	<0.01	0	0	<0.01	0.02	0	0	0	0.07	0	<0.01	<0.01	0	0.01	0

**Parvularcula**	0	0	0	0	0	0	0	0	0	0	0	0	<0.01	<0.01	0	0
**Pasteurella**	0	0	0	0	0	<0.01	0	0	0	0	0	0	<0.01	<0.01	<0.01	<0.01
**Pasteuriaceae Incertae Sedis**	0	0	0	0	0	<0.01	0	0	0	0	0	0	0	0	0	0
**Pectobacterium**	0	0	0	<0.01	0	<0.01	0	0	0	0	0	0	0	<0.01	0	0
**Pelobacter**	0	0	0	0	0	0	0	0	0	0	0	0	0	<0.01	0	0
**Pelomonas**	0.02	<0.01	0	0.04	0.01	0.05	0	<0.01	<0.01	<0.01	0	<0.01	0.01	0	0.07	0
**Peptoniphilus**	0	0	0	0	<0.01	0	0	0	0	0	0	0	0	0	0	0
**Peredibacter**	0	0	0	0	0	<0.01	0	0	0	0	0	0	0	<0.01	<0.01	0
**Persicobacter**	0	0	0	0	<0.01	0	0	0	0	0	0	0	<0.01	<0.01	0.02	<0.01
**Petrobacter**	0	0	0	<0.01	0	0	0	0	0	0	0	0	0	0	0	0
**Phaeobacter**	0	0	0	0	0	1.13	0	0	0	0	0	0	0	0	0	0
**Phenylobacterium**	0	0	0	0	<0.01	0	0	<0.01	0	0	0	<0.01	0.05	<0.01	0.03	<0.01
**Photobacterium**	0	0	0	0.01	0	0.02	0	0	0	0	0	0	<0.01	0.06	0.03	0.02
**Phyllobacterium**	0	0	0	<0.01	0	0	0	0	0	0	0	0	0	<0.01	0.03	0
**Pirellula**	0	0	0	0	0	<0.01	0	0	0	0	0	0	<0.01	<0.01	0	<0.01
**Piscirickettsia**	0	0	0	<0.01	0	0	0	0	0	0	0	0	<0.01	0	0	0
**Planctomyces**	0	0	0	0	0	0.02	0	0	0	0	0	0	<0.01	<0.01	0.01	<0.01
**Planomicrobium**	0	0	0	0	0.01	0	0	0	0	0	0	0	0	0	0	0
**Polaribacter**	0	0	0.39	0.02	<0.01	0.03	0	0	0	0	0	0	<0.01	0.02	<0.01	<0.01
**Polyangiaceae**	0	0	0	0	0.01	<0.01	0	0.01	0.02	<0.01	0	0	0.04	<0.01	0.01	0.02
**Polynucleobacter**	0	0	0	0	0	0	0	<0.01	0	0	0	0	0	0	0	0
**Pontibacter**	0	0	0	<0.01	0	0	0	0	0	0	0	0	0	<0.01	0	0
**Porphyromonas**	0	0	0	0	0	<0.01	0	0	0	0	0	0	0	0	0	0
**Prevotella**	0	0	0	0	0	0	0	0	0	0	<0.01	0	0	<0.01	0.01	0
**Propionibacterineae**	0.08	0.02	<0.01	0.25	0.21	0.15	<0.01	0	0.01	0.01	0	<0.01	0.01	<0.01	0.39	<0.01
**Propionigenium**	0	0	0	0	0	<0.01	0	0	0	0	0	0	0.02	<0.01	0	<0.01
**Propionivibrio**	0	0	0	0	0	<0.01	0	0	0	0	0	0	0	0	0	0
**Proteus**	0	0	0	<0.01	<0.01	<0.01	0	0	0	0	0	0	0	<0.01	0	0
**Providencia**	0	0	0	0	0	0	0	0	0	0	0	0	0	<0.01	0	0
**Pseudidiomarina**	0	0	0	0	0	0	0	0	0	0	0	0	0.03	0	0	0
**Pseudoalteromonas**	0	0	0	6.45	0.06	0.4	0	0	0	0	0	<0.01	0.07	0.02	0.14	0.07

**Pseudomonas**	0	0	0.01	0.02	<0.01	0.45	0	0	<0.01	0.02	0.36	0.03	0.42	<0.01	15.3	0.23
**Pseudonocardineae**	0	0	0	0	0.01	<0.01	0	0	0	0	0	0	0	<0.01	0	0
**Pseudospirillum**	0	0	0	0	0	0	0	0	0	0	0	0	0	0	0.01	0
**Pseudovibrio**	0	0	0	0	0	0	0.01	0	0.12	0	0	0	<0.01	<0.01	0	0
**Pseudoxanthomonas**	0.01	0	0	0	0	0	0	0	0	0	0	0	0	0	0.08	0
**Psychrobacter**	0	0	0	0.28	0.03	0.04	0	0	0	0	<0.01	<0.01	0.02	0.01	0.33	<0.01
**Psychroflexus**	0	0	0	0	0	0	0	0	0	0	0	0	0	<0.01	0	0
**Psychromonas**	0	0	0	<0.01	0	0	0	0	0	0	0	0	0	<0.01	0.01	0
**Ralstonia**	0.12	<0.01	0.16	0.06	0.12	0.05	0.01	<0.01	0.09	0.06	0.01	<0.01	0.04	0	0.95	<0.01
**Reichenbachiella**	0	0	0	0.33	0.02	0.15	0	0	0	0	0	0	0.02	0.04	0.02	0.03
**Rheinheimera**	0	0	0	0.01	0.03	0.11	0	0	0	0	0	0	0	<0.01	0.02	<0.01
**Rhizobium**	0	<0.01	0	0	0.01	<0.01	0	<0.01	0	0	0	0	0	<0.01	0	0
**Rhodanobacter**	0	0	0	0	0	0	0	0	0	0	0	<0.01	0	0	0.01	0
**Rhodobacter**	0	<0.01	0	0.03	0.02	0.04	0	<0.01	0	0.93	0	0.05	<0.01	0	0	0
**Rhodobium**	0	0	0	0	0	<0.01	0	<0.01	0	0	0	0	0	0	0	0
**Rhodomicrobium**	0	0	0	0	0	<0.01	0	0	0	0	0	0	0	0	0	0
**Rhodoplanes**	0	<0.01	0	0	<0.01	0	0	<0.01	0	0	0	0	0	<0.01	0	0
**Rhodothermus**	0	0	0	0	0	0	0	0	0	0	0	0	<0.01	<0.01	0	0
**Rhodovulum**	0	0	0	0	0	0.04	0	0	0	0	0	0	0.03	<0.01	0	0.02
**Rickettsiella**	0	0	0	0	0	0	0	0	0	0	<0.01	0	0	0	0	0
**Robiginitalea**	0	<0.01	0	0	0	0.02	0	0	0	0	0	0	0.03	0.01	<0.01	0.01
**Roseicyclus**	0	<0.01	0	0	0	0	0	0	0	0	0	0	0	0	0	0
**Roseivirga**	0	0	0	0	0	<0.01	0	0	0	0	0	0	0	0	0	0
**Roseivivax**	0	0	0	<0.01	0	0	0	0	0	0	0	0	0	0	0	0
**Roseomonas**	0	0	0	0	0	0	0	0	0	0	0	0	<0.01	0	0	0
**Roseospirillum**	0	0	0	0	0	<0.01	0	0	0	0	0	0	0	0	<0.01	<0.01
**Roseovarius**	0.01	<0.01	0	0	<0.01	0.04	0.03	0.03	0.04	0	0	0	0.35	0.5	0.18	0.64
**Rubritalea**	0	0	0	<0.01	0	0.13	0	0	0	0	0	0	0.11	0.03	0	0.03
**Rubrobacterineae**	0	0	<0.01	0	0	0	0	<0.01	0	0	0	0	0	<0.01	<0.01	0
**Runella**	0	0	0	0	0.02	0.01	0	0	0	0	0	0	0	0	0	0
**Saccharospirillum**	0	0	0	<0.01	0	0	0	0	0	0	0	0	0.01	<0.01	0.01	0

**Salicola**	0	0	0	0	0	0	0	0	0	0	0	0	0	<0.01	0	0
**Salinibacter**	0	0	0	<0.01	0	0	0	0	0	0	0	0	0	0	0	0
**Salinicoccus**	0	<0.01	0	0	0	0	0	0	0	0	0	0	0	0	0	0
**Salinisphaera**	0	0	0	<0.01	0.02	0.01	0	0	0	0	0	0	0	<0.01	0	0
**Salinivibrio**	0	0	0	0	0	<0.01	0	0	0	0	0	0	0	0	0	0
**Sandarakinotalea**	0	0	0	0	0	0.03	0	0	0	0	0	0	0	0	0	0
**Saprospira**	0	0	0	<0.01	0	0	0	0	0	0	0	0	0	0	0	<0.01
**Sediminicola**	0	0	0	<0.01	0.01	<0.01	0	0	0	0	0	0	0.06	0.21	0.07	0.07
**Serratia**	0	0	0	0	0	0.01	0	<0.01	<0.01	0.02	0.01	<0.01	0	0	0	0
**Shewanella**	0	0	0	0.14	0.02	0.03	0	0	0	0	0	0	<0.01	0.01	0	<0.01
**Shigella**	0	0	0	0	0	0	0	0	0.01	0	0	0	0	0	0	0
**Shinella**	0	0	0	<0.01	0	<0.01	0	0	0	0	0	0	0	0	0	0
**Silicibacter**	0	<0.01	<0.01	0.1	0.12	0.73	<0.01	0	<0.01	0.03	2.23	0.02	0.18	0.04	0.07	0.12
**Simonsiella**	0	<0.01	0	0	0	0	0	0	0	0	0	0	0	0	0	0
**Sphingobium**	0.02	<0.01	0.01	0.43	0.02	0.01	0	0.05	0	0.02	<0.01	0.04	0.07	0.02	0.11	<0.01
**Sphingomonas**	0.02	0	0.03	0.09	0.03	0.01	0	<0.01	0.01	0	0	0	0.17	0.05	0.02	0.03
**Sphingopyxis**	0	0	0	0	<0.01	<0.01	0	0	0	0	0	0	0	<0.01	0	<0.01
**Spirochaeta**	0	0	0	0	0	0	0	0	0	0	0	0	0	<0.01	<0.01	0
**Staphylococcus**	0	<0.01	0.01	0.08	0.01	0.02	0	0.01	0	0	0	0	0	<0.01	0.02	<0.01
**Stappia**	0	0	0	0	0	0.08	0	<0.01	0	0	0	0	<0.01	<0.01	0	0
**Stella**	0	0	0	0	0	<0.01	0	0	0	0	0	0	<0.01	0	0	0
**Stenotrophomonas**	0	<0.01	0	0	0.01	0	0	0	<0.01	<0.01	0	<0.01	0	<0.01	0.1	0
**Streptococcus**	0	<0.01	0	0.03	0.01	<0.01	0	0	0	<0.01	0	0	<0.01	<0.01	0.06	0
**Streptomycineae**	0	0	0	0	0	0.03	0	0	0	0	0	0	0	0	0	0
**Subdivision 5_genera_incertae_sedis**	0	0	0	0	0	<0.01	0	0	0	0	0	0	<0.01	<0.01	0	<0.01
**Sulfitobacter**	0	0	0	0.03	0.01	0.12	0	0.01	0	0	0	0	0.04	0.01	0.02	0.04
**Sulfurovum**	0.01	0	0	0	0	0	0	0	0	0	<0.01	0	<0.01	0	0	0
**Suttonella**	0	0	0	0	0.01	<0.01	0	0	0	0	0	0	0	<0.01	0.03	0
**Tenacibaculum**	0	0	0	0.06	<0.01	0.06	0	<0.01	0	0	0	0	0.04	0.02	0.02	0.03
**Tepidimonas**	0	<0.01	0	<0.01	0	<0.01	0	0	0	0	0	0	<0.01	0	0.04	0

**Terasakiella**	0	0	0	0	0	<0.01	0	0	0	0	0	0	0	<0.01	0	0
**Teredinibacter**	0	0	0	<0.01	0	<0.01	0	0	0	0	0	0	<0.01	<0.01	0	0
**Thalassomonas**	0	0	0	0.48	0	0.05	0	0	0	0	0	0	0.04	0.01	0	0.02
**Thauera**	0	0	0	<0.01	0	0	0	0	0	0	0	0	0	0	0	0
**Thermoanaerobacterium**	0	<0.01	0	0	0	0	0	0	0	0	0	0	0	0	0	0
**Thermus**	0	0	0	0	0	0	0	0	0	0	0	<0.01	0	0	0	0
**Thioalkalispira**	0	0	0	0	0	0	0	<0.01	0	0	0	0	<0.01	0	0	0
**Thioalkalivibrio**	0.13	0.1	0.27	2.37	4.94	1.69	0	0.04	0	0.41	0.7	0.34	0	<0.01	0	0
**Thiomicrospira**	0	0	0	<0.01	0	0.04	0	0	0	0	0	0	0.02	0.02	0.02	0.03
**Thiothrix**	0	0	0	0.02	0.02	<0.01	0	0	0	0	0	0	0	<0.01	0	0
**Truepera**	0	0	0	0	0	<0.01	0	0	0	0	0	0	<0.01	<0.01	0	0
**Verrucomicrobiaceae_genera_incertae_sedis**	0.01	0	0.02	<0.01	0.01	0.07	0.03	0	0.02	0	0	0	0.18	0.27	0.08	0.37
**Vibrio**	0	0	0	0.15	0.03	0.23	0	0	<0.01	0	0	0.03	0.11	0.09	0.11	0.11
**Winogradskyella**	0	<0.01	0	0.05	0.03	0.14	0	0	0	0	0	0	0.13	0.26	0.1	0.3
**Woodsholea**	0	0	0	0	0	<0.01	0	0	0	0	0	0	0	<0.01	0	0
**Xiphinematobacteriaceae_genera_incertae_sedis**	0	0	0	0	<0.01	<0.01	0	0	0	0	0	0	<0.01	0	0.01	<0.01
**Zymobacter**	0	0	0	0	0	<0.01	0	0	0	0	0	0	0	0	0	0
**genus_BTH**	39.7	43.2	40.5	23	33.8	20.5	42.5	35.4	19.8	24.1	34.4	29.7	10.7	12.1	8.01	10.9
**genus_NA**	44.6	41.7	44.1	29.8	41.3	22.2	55.2	62	77.3	56.3	45.5	60.8	45.1	49.8	42.1	53.1
**Bacillaceae 1**	0.03	0	0	0.06	0.03	11.1	0	0	<0.01	<0.01	0	0	1.11	0.27	1.22	0.22
**Bacillaceae 2**	0	0	0	0.01	0	0.01	0	0	0	0	0	0	0	<0.01	0.01	0
**Carnobacteriaceae 1**	0	0	0	0	0	0	0	0	0	0	0	0	0	0	0.01	0
**Clostridiaceae 1**	0	0	0	0	0	0	0	0	0	0	0	0	0	<0.01	<0.01	0.02
**Lachnospiraceae Incertae Sedis**	0.02	0	0	0	0	0	0	<0.01	0	0	0	0	0	<0.01	0	0
**Paenibacillaceae 1**	0	0	0	0	0	0.01	0	0	0	0	0	0	<0.01	0	0	<0.01
**Peptostreptococcaceae Incertae Sedis**	0	0	0	0	0	0	0	0	0	0	0	0	<0.01	<0.01	0	0
**Ruminococcaceae Incertae Sedis**	0	<0.01	0	0	0	0	0	0	0	0	0	0	0	0	0	0
**Sporolactobacillaceae Incertae**	0	0	0	0	0	<0.01	0	0	0	0	0	0	0	0	0	0

**Sedis**																
**unclassified_"Lachnospiraceae"**	0	0	0	0	0	0	0	<0.01	0	0.01	0	0	0	<0.01	0	<0.01
**unclass_"Ruminococcaceae"**	0	0	0	0	0	0	0	0	0	0	0	0	0	0	0	<0.01
**unclassified_Actinomycetales**	0.01	<0.01	0.02	<0.01	0.03	0.04	<0.01	<0.01	<0.01	0	0	0	0.11	0.11	0.08	0.13
**unclassified_Alteromonadaceae**	0	0	0	0	<0.01	0.08	0	0	0	0	0	0	0	0	0	<0.01
**unclass_Bacteriovoracaceae**	0	0	0	0	0	0	0	0	0	0	0	0	<0.01	0	0.01	0
**unclassified_Caulobacteraceae**	0.01	0	0	0	0	0	0	<0.01	0	0	0	0	0	0	<0.01	0
**unclassified_Chromatiaceae**	0	0	0	0	0	0	0	0	0	0	0	0	0	<0.01	0	0.01
**unclassified_Crenotrichaceae**	0	<0.01	0	0	0.02	0	0	0.06	<0.01	6.86	6.43	3.19	0	<0.01	0.02	0
**unclassified_Cryomorphaceae**	0	0	0	<0.01	<0.01	<0.01	<0.01	<0.01	0.01	0	<0.01	0	0.06	0.26	0.03	0.2
**unclass_Desulfobacteraceae**	0	0	0	0	<0.01	0.04	0	0	0	0	0	<0.01	0.02	0.04	0	0.04
**unclassified_Desulfobulbaceae**	0	<0.01	0	0	<0.01	0.02	<0.01	0	0	0	0	0	0.07	0.04	0.01	0.05
**uncl_Ectothiorhodospiraceae**	2.12	1.29	0.86	0.47	0.29	0.22	0.3	0.37	0.56	0.58	0.16	0.04	0.11	0.08	0.05	0.27
**unclassified_Enterobacteriaceae**	0.01	<0.01	0.01	<0.01	0.02	0.01	<0.01	0	0	<0.01	0	0	0	<0.01	0.06	0
**unclassified_Flavobacteriaceae**	0.02	0.04	0.06	0.22	0.1	0.58	0.15	0.13	0.13	0.01	0.02	0.06	3	8.55	1.78	7.2
**unclassified_Flexibacteraceae**	0.02	<0.01	0	0.03	0.05	0.18	<0.01	0.02	0.01	0	0.16	<0.01	0.22	0.62	0.12	0.68
**unclassified_Halomonadaceae**	0	0	0	0	0.03	0	0	0	0	0	0	0	0	0	<0.01	0
**unclass_Hyphomicrobiaceae**	0	0	0	0	0	<0.01	0	0	0	0	0	0	<0.01	0	0	<0.01
**unclassified_Incertae Sedis XII**	0	0	0	0	0	0	0	0	0	0	0	0	0	0	0	<0.01
**unclassified_Incertae sedis 7**	0	0	0	<0.01	0	0.02	0	0	0	0	0	0	0.02	<0.01	0.04	<0.01
**unclassified_Leptospiraceae**	0	0	0	0	0	0	0	0	0	0	0	0	0	<0.01	0	0
**unclassified_Moraxellaceae**	0	0	0	0	0	<0.01	0	0	0	0	0	0	0	0	0	0
**unclassified_Neisseriaceae**	0	0	0	<0.01	0	0	0	0	0	0	0	0	0	0	0	0
**unclassified_Oceanospirillaceae**	0	0	0	0.12	0	0	0	0	0	0	0	0	<0.01	<0.01	0	0
**unclassified_Phyllobacteriaceae**	0	<0.01	0	0	0	0.02	0	<0.01	0	0	0	0	0.01	<0.01	<0.01	<0.01
**unclass_Planctomycetaceae**	0	<0.01	0	0.05	0.07	0.3	<0.01	0	0.01	0	<0.01	0	0.25	0.21	0.28	0.29
**unclass_Pseudomonadaceae**	0	0	0	0	0	0	0	0	0	0	0	0	0	0	0.04	0
**unclassified_Rhodobacteraceae**	0.09	0.04	0.17	1.37	3.58	0.66	0.87	0.6	0.86	3.92	0.46	0.95	7.6	8.69	4.17	10.4
**unclassified_Rhodobiaceae**	0	0	0	0	0	0	0	<0.01	0	0	0	0	0	0	0	0
**unclassified_Rhodocyclaceae**	0.01	0	0	<0.01	0	<0.01	0	0	0	0	0	0	0	0	0.01	0
**unclassified_Rhodospirillaceae**	0	0	0	0	0	0	0	0	0	0.05	0	0	0	0	0	0

**unclassified_Rikenellaceae**	0	0	0	0	0	0	0	0	0	0	0	0	0	0	<0.01	0
**unclassified_Saprospiraceae**	0	0	0	0	<0.01	0.07	<0.01	0.01	0	0	0	0	0.04	0.04	0.03	0.03
**unclass_Sphingomonadaceae**	0	0	0	<0.01	0	<0.01	0	0	0	0	0	0	<0.01	0	0	0
**unclassified_Syntrophaceae**	0	0.02	0.1	0.05	0.01	<0.01	0	0.03	<0.01	0.46	0.19	0.09	<0.01	0	0	0
**unclass_Verrucomicrobiaceae**	0	0	0	0	0	<0.01	0	0	0	0	0	0	0	<0.01	0	<0.01
**unclassified_Vibrionaceae**	0	0	0	0	0	0	0	0	0	0	0	0	<0.01	<0.01	0	0
**unclass_Xanthomonadaceae**	0	0	0	0	0.01	0	0	0	0	0	0	0	0	0	0	0

Table S2 Taxonomic assignments of V6 sequence tags from sponge and seawater samples at the phylum level. Relative abundances (in %) are denoted. Sample numbers: 1-3, *R. odorabile*; 4-6, *R. odorabile* larvae; 7-9, *I. basta*; 10-12, *I. ramosa*; 13-16, seawater.

	**1**	**2**	**3**	**4**	**5**	**6**	**7**	**8**	**9**	**10**	**11**	**12**	**13**	**14**	**15**	**16**
**Acidobacteria**	9.79	10.6	10.3	2.8	5.77	1.49	0.04	0.57	0.06	12.6	4.46	5.04	0.16	0.07	0.11	0.22
**Actinobacteria**	8.2	8.93	11	6.64	13.4	3.09	0.06	0.35	0.08	8.23	10.9	15.3	1.22	0.94	2.21	1.45
**Aquificae**	0.33	0.42	0.39	0.05	0.27	0.01	0	0	0	0	0	0	0	0	0	0
**BRC1**	0	0	0	0	0	<0.01	0	0	0	0	0	0	0	0	0	0
**Bacteroidetes**	0.27	0.35	2.04	2.25	0.95	5.02	0.52	0.44	0.39	7.03	6.81	3.63	11.8	17.4	4.51	13.5
**Chlamydiae**	0	0	0	<0.01	0.03	0.02	0	<0.01	0	0.01	0	0	0.03	0.03	0.01	0.02
**Chlorobi**	0	0	0	0	0	0	0	0	0	0	0	0	<0.01	0	0	<0.01
**Chloroflexi**	11.2	11.7	11.7	3.8	7.37	1.06	0.02	0.3	0.01	4.53	6.5	4.87	0.11	0.09	0.05	0.11
**Chloroplasts**	0.07	0.02	0.08	26.8	6.47	32.4	0.64	0.44	0.58	0.03	0.03	0.03	21.7	15.6	21.3	12.3
**Cyanobacteria**	0.71	0.17	0.6	7.54	2.25	13.7	1.34	1.07	1.22	0.23	0.18	0.26	10.6	12.8	13.9	16.1
**Deferribacteres**	0.22	<0.01	0.02	<0.01	0.02	<0.01	0	0	0	0.29	0.24	0.12	0	0.02	0	0.03
**Deinococcus-Thermus**	0.19	0.11	0	0.05	0.09	0.03	0	<0.01	0	0	0	<0.01	0.03	0.01	0	0.02
**Fibrobacteres**	0	0	0	0	0	<0.01	0	0	0	0	0	0	0	0	0	0
**Firmicutes**	6.31	5	4.65	2.13	4.54	12.8	0.05	0.18	0.06	3.79	5.36	4.26	1.89	1.37	2.12	0.98
**Fusobacteria**	0	0	0	0	0	0.02	0	0	0	0	0	0	0.03	0.02	0.01	0.01
**Gemmatimonadetes**	4.35	5.68	4.94	3.4	6.92	0.68	0	0.32	0.03	7.94	4.59	4.97	0.03	<0.01	0.02	0.02
**Lentisphaerae**	0	0	0	0.23	0.12	0.79	0	0	<0.01	0	0	0	0.28	0.23	0.12	0.22
**Mitochondria**	0	0	0	0	0.01	<0.01	0	0	0	<0.01	0	0	0.04	<0.01	0.04	0.03
**Nitrospira**	2.44	3.07	2.3	2.12	0.69	0.18	0	0.02	<0.01	0.83	3.44	0.44	<0.01	0.02	0.04	<0.01
**OD1**	0	0	0	0	0	0	0	0	0	0	0	0	0	0.01	0.02	0.01
**OP10**	0	0	0	0	0	0	0	0	0	0	0	0	0	<0.01	0	<0.01
**OP11**	0	0	0	0	0	0	0	0	0	0	0	0	<0.01	<0.01	0.01	<0.01
**Planctomycetes**	0.03	0.05	0.02	0.08	0.17	0.61	0.01	<0.01	0.01	0.01	0.01	<0.01	0.66	0.46	0.5	0.59
**Poribacteria**	4.08	3.28	4.24	1.17	3.5	0.27	0	0.06	0.01	1.45	3.08	2.01	0	0	0.02	0
**Proteobacteria**	44.1	44.4	39	38.3	38.8	25.2	66.8	73.4	81.9	35.4	32.7	31.8	47.8	47.5	52.3	50.9
**Spirochaetes**	0.13	0.29	0.15	0.02	0.08	0.04	<0.01	<0.01	<0.01	0.04	0.4	0.05	0.02	0.02	0.03	0.02
**TM7**	0	0	0	0	<0.01	0.01	0	<0.01	0	0	0	0	0.06	0.04	0	0.05

**Tenericutes**	0	<0.01	0	<0.01	0	<0.01	0	0	0	0	0	0	0.1	0.04	0.01	0.03
**Thermodesulfobacteria**	0	0	0	0	0	0	0	0	0	0	0	0	0	<0.01	0	0
**Thermomicrobia**	0	0	0	0	0	0	0	0	0	0	0	0	<0.01	0	0	0
**Thermotogae**	0.26	0.32	0.35	0.07	0.21	0	0	0	0	0.08	0	0	0	0	0	0
**Verrucomicrobia**	0.01	<0.01	0.03	0.22	0.2	1.09	0.1	0.09	0.1	0.01	0.02	0	1.78	1.96	1.02	2.18
**WS3**	0	<0.01	0	0	0.01	0.01	0	0	0	0	0	0	<0.01	<0.01	0	0.02
**SC 26**	4.38	2.21	5.15	0.94	2.57	0.21	0	0.12	<0.01	3.31	3.47	6.25	0	<0.01	<0.01	<0.01
**phylum_BTH**	0.66	0.44	0.47	0.31	0.46	0.47	30.3	22.5	15.4	0.77	2.87	4.66	1.55	1.23	1.54	1.07
**phylum_NA**	2.24	2.97	2.66	1.12	5.15	0.71	0.05	0.1	0.09	13.4	15	16.3	0.06	0.06	0.02	0.07

Table S3 Taxonomic assignments of V6 sequence tags from sponge and seawater samples at the family level. Relative abundances (in %) are denoted. Sample numbers: 1-3, *R. odorabile*; 4-6, R. odorabile larvae; 7-9, I. basta; 10-12, I. ramose; 13-16, seawater.

	**1**	**2**	**3**	**4**	**5**	**6**	**7**	**8**	**9**	**10**	**11**	**12**	**13**	**14**	**15**	**16**
**Acetobacteraceae**	0	0	0	0	0	<0.01	0	0	0	0,02	0	0	0,02	0	0	0
**Acholeplasmataceae**	0	0	0	<0.01	0	0	0	0	0	0	0	0	0	<0.01	0	<0.01
**Acidimicrobiales**	0,02	0,01	0,08	0	0,05	0	0	0	0	0	0	0	0	0	0	<0.01
**Acidithiobacillaceae**	0	0	0	<0.01	0	0	0	0	0	0	0	0	0	0	0	0
**Acidobacteriaceae**	8,89	9,63	10,1	2,49	4,62	1,35	0,02	0,51	0,04	10,3	3,39	3,11	0,12	0,04	0,08	0,17
**Actinomycetales**	0,16	0,08	0,04	1,25	0,66	1,3	<0.01	<0.01	0,04	0,03	0,01	<0.01	0,5	0,15	0,91	0,15
**Aeromonadaceae**	0	0	0	0	0	0,61	0	0	0	0	0	0	<0.01	0	0	0
**Alcaligenaceae**	0	0	0	0	0	0	0	0,08	0,04	0,01	0,01	0,02	<0.01	0	0,02	0
**Alcanivoraceae**	0	0	<0.01	<0.01	0,17	0,24	<0.01	0	0	0	0	0	<0.01	<0.01	0,02	0
**Alteromonadaceae**	0	0	0	0,74	0,07	1,02	0	<0.01	<0.01	0	0	0	0,18	0,05	0,03	0,08
**Aurantimonadaceae**	0	0	0	0	0	0	0	0	0	0	0	0	0	<0.01	0	0
**Bacillaceae**	0,03	0	0	0,08	0,03	11,2	0	0	<0.01	<0.01	0	0	1,11	0,28	1,25	0,23
**Bacteriovoracaceae**	0,01	0,01	0	<0.01	0	0,01	0	0	<0.01	0,14	0,05	0,06	0,02	0,03	0,04	0,01
**Bacteroidaceae**	0	0	0	<0.01	0	0	0	0	0	0	0	0	0	<0.01	0,04	0
**Bartonellaceae**	0	0	0	0	0	0	0	<0.01	0	0	0	0	0	0	0	<0.01
**Bdellovibrionaceae**	0	0	0,19	<0.01	0	0	<0.01	0	0	0	0	0	0	<0.01	0	0
**Beijerinckiaceae**	0	0	0	0	0	0	0	0	0	0	0	0	0	<0.01	0	0
**Bifidobacteriales**	0	0	0	<0.01	0	0	0	0	0	0	0	0	<0.01	0	<0.01	0
**Bradyrhizobiaceae**	0	0	0	0,04	<0.01	<0.01	0	0,01	0	0,03	<0.01	0	<0.01	<0.01	0,08	0
**Brucellaceae**	0	0	0	0	0	<0.01	0	0	0	0,01	0	0	<0.01	<0.01	0	0
**Burkholderiaceae**	0,12	<0.01	0,17	0,07	0,13	0,05	0,02	0,02	0,1	0,07	0,02	<0.01	0,06	0,04	1,02	0,05
**Caldilineales**	2,61	2,29	2,35	1,66	3,16	0,45	0	0,17	<0.01	3,15	4,81	3,7	0,05	0,04	0,03	0,05
**Campylobacteraceae**	0	0	0	0	0	0	0	0	0	0	0	0	<0.01	<0.01	0	<0.01
**Cardiobacteriaceae**	0	0	0	<0.01	0,01	<0.01	0	<0.01	0	0	0	0	0	<0.01	0,03	<0.01
**Carnobacteriaceae**	0	0	0	0	0	0	0	0	0	0	0	0	0	<0.01	0,01	0
**Caulobacteraceae**	0,02	0	0	0,03	0,02	<0.01	<0.01	0,05	0	0,05	0,01	0,01	0,13	0,02	0,04	<0.01
**Chloroplasts**	0,07	0,02	0,08	26,5	6,4	31,8	0,63	0,44	0,56	0,03	0,03	0,03	21,3	15,4	21	12,1

**Chromatiaceae**	0	0,03	0,02	0,02	0,03	0,11	0	0	0	0	0	0	<0.01	0,02	0,02	0,04
**Clostridiaceae**	0	0	0	0	0	0	0	0	0	0	0	0	<0.01	<0.01	<0.01	0,02
**Colwelliaceae**	0	<0.01	0	0,9	<0.01	0,74	<0.01	0	0	0	0	0	0,09	0,03	0,04	0,05
**Comamonadaceae**	0,31	0,04	0,13	0,79	0,29	0,37	0,04	0,08	0,15	0,05	0,06	0,08	0,49	0,07	0,47	0,03
**Coxiellaceae**	0,02	1,1	0,39	0,04	0,03	0,08	<0.01	<0.01	0	0,17	0,2	1,65	0,07	0,08	0,03	0,07
**Crenotrichaceae**	0	<0.01	0,02	0,19	0,13	0,62	<0.01	0,08	0,02	6,87	6,47	3,43	0,04	0,07	0,04	0,06
**Cryomorphaceae**	0	0	<0.01	0,09	0,03	0,2	0,01	0,02	0,03	0	0,01	<0.01	0,43	1,05	0,26	0,72
**Cystobacterineae**	1,29	1,17	1,03	0,18	0,4	0,03	0	0	0	0,05	0,09	0	0	<0.01	0	0
**Deferribacteraceae**	0	0	0,01	0	0	0	0	0	0	<0.01	0	0,04	0	0	0	0
**Deinococcaceae**	0	0	0	0	0	<0.01	0	0	0	0	0	0	0	0	0	0
**Desulfarculaceae**	0	0	0	0	0	0	0	0	0	0	0	0	0	0	0	<0.01
**Desulfobacteraceae**	0	0	0	<0.01	<0.01	0,06	<0.01	0	0	0	0	<0.01	0,02	0,06	<0.01	0,08
**Desulfobulbaceae**	0	<0.01	0	<0.01	0,02	0,03	<0.01	0	0	0	0	0	0,1	0,05	0,02	0,07
**Desulfohalobiaceae**	0	0	0	0	0	0	0	0	0	0	0	0	0	<0.01	0	0
**Desulfomicrobiaceae**	0	0	0	0	0	<0.01	0	0	0	0	0	0	0	0	0,01	0
**Desulfovibrionaceae**	0	0	0	0	0	0,01	0	0	0	0	0	0	0,04	0,02	0,02	0,02
**Desulfuromonaceae**	0	0	0	<0.01	<0.01	0	0	0	0	0	0	0	0	<0.01	0	0
**Ectothiorhodospiraceae**	5,88	4,33	6,14	5,71	7,05	2,18	0,3	0,44	0,59	2,78	2,19	1,82	0,19	0,12	0,06	0,41
**Enterobacteriaceae**	0,06	<0.01	0,01	0,6	0,05	0,34	<0.01	0,04	0,1	0,12	0,04	0,07	0,26	0,12	0,14	0,11
**Enterococcaceae**	0	<0.01	0	0	0	0	0	0	0	0	0	0	0	<0.01	0,02	0
**Entomoplasmataceae**	0	<0.01	0	<0.01	0	0	0	0	0	0	0	0	0	<0.01	0	0
**Erysipelotrichaceae**	0	0	0	<0.01	0	0,01	<0.01	<0.01	0,03	0	0	<0.01	0,2	0,59	0,06	0,28
**Ferrimonidaceae**	0	0	0	<0.01	0	0,02	0	0	0	0	0	0	0,03	<0.01	<0.01	0,02
**Flammeovirgaceae**	0	0	0	0,02	0,01	0,08	0	0	0	0	0	0	0,09	0,05	0,06	0,04
**Flavobacteriaceae**	0,12	0,06	0,51	0,79	0,42	1,81	0,19	0,15	0,2	0,05	0,06	0,11	9,41	11,7	2,86	9,14
**Flexibacteraceae**	0,02	<0.01	0	0,48	0,14	0,87	<0.01	0,06	0,01	0,01	0,17	<0.01	0,35	0,78	0,18	0,76
**Francisellaceae**	0	0	0	0,07	0,01	0,07	0	<0.01	0	0	0	0	0,02	<0.01	<0.01	0,01
**Fusobacteriaceae**	0	0	0	0	0	0,02	0	0	0	0	0	0	0,02	<0.01	0	0,01
**Gemmatimonadaceae**	0,06	0	0	0	0	0,02	0	0	0	0	0	0	0	<0.01	0	<0.01
**GpI**	0	0	0	0	<0.01	0,01	0	0	0	0	0	0	0	<0.01	0	0
**GpIIa**	0,71	0,16	0,6	0,12	0,12	0,36	1,34	0,69	1,21	0,1	0,04	0,14	10,4	12,7	13,8	15,8

**GpIIb**	0	0	0	0	<0.01	0	0	0	0	0	0	0	0	0	0	0
**GpIV**	0	0	0	0	0	0,05	0	0,18	0	0	0	0	<0.01	<0.01	0	0
**GpIX**	0	0	0	0	0	0,02	0	0	0	0	0	0	0	0	0	0
**GpVII**	0	0	0	0	0	<0.01	0	0	0	0	0	0	0	0	<0.01	0
**GpVIII**	0	0	0	0	0	0	0	0,01	0	0	0	0	<0.01	<0.01	0	0
**GpX**	0	0	0	0	0	<0.01	0	0	0	0	0	0	<0.01	<0.01	0	0,01
**GpXI**	0	0	0	0	0	<0.01	0	0	0	0	0	0	0	<0.01	0	0
**GpXII**	0	0	0	7,34	2,1	12,9	0	0	0	0	0	0	0,07	0,03	<0.01	0,16
**GpXIII**	0	0	0	0	0	0,08	0	0	0	0	0	0	<0.01	<0.01	0,05	<0.01
**Hahellaceae**	0	0	0	0	0	0,01	0	0	0	0	0	0	<0.01	0	0	0
**Halomonadaceae**	0,12	0,3	0,57	0,18	0,49	0,84	0	<0.01	0,02	0,16	0,04	1,06	<0.01	<0.01	0,03	<0.01
**Halothiobacillaceae**	0	0,01	0	<0.01	0,01	0,02	0	0,02	0	0	0	0	<0.01	<0.01	<0.01	<0.01
**Helicobacteraceae**	0,01	0	<0.01	0	0	<0.01	0	0	0	0,01	<0.01	<0.01	<0.01	<0.01	0	0
**Herpetosiphonaceae**	0	0	0	0	0	<0.01	0	0	0	0	0	0	0	0	0	0
**Hydrogenimonaceae**	0	0	0	0	0	<0.01	0	<0.01	0	0	0	0	0	0	0	0
**Hydrogenophilaceae**	0	0	0	<0.01	0	<0.01	0	0	0	0	0	0	0	0	0	0
**Hyphomicrobiaceae**	0	<0.01	0	0,03	0,03	0,02	0,07	0,02	0	0	0	<0.01	0,03	<0.01	0,01	0,03
**Idiomarinaceae**	0	0	0	0,08	0,04	0,44	0	0	0	0	0	0	0,07	<0.01	0,03	0,02
**Incertae sedis 11**	0	0	0	0	0	0	0	0	0	0	0	0	<0.01	<0.01	0,01	0
**Incertae sedis 3**	0	0	0	0	0	0	0	0	0	0	0	0	0	0,01	0	0,01
**Incertae sedis 4**	0	0	0	<0.01	0,02	<0.01	0	0	0	0	0	0	0,04	<0.01	0	<0.01
**Incertae sedis 5**	0,02	<0.01	0,02	0,23	0,2	0,46	0,02	0,02	0,01	0,02	<0.01	0,01	0,03	<0.01	0,16	0
**Incertae sedis 6**	0	0	0	<0.01	0	0	0	0	0	0	0	0	0	0	0	0
**Incertae sedis 7**	0	0	0,01	0,17	0,09	0,97	0,06	1,15	0,04	0	0,01	0	0,27	0,16	0,41	0,16
**Incertae Sedis III**	0	<0.01	0	0	0	0	0	0	0	0	0	0	0	0	0	0
**Incertae Sedis XI**	0	0	0	0	<0.01	<0.01	0	0	0	0	0	0	0	0	0,01	0
**Incertae Sedis XII**	0	0	0	<0.01	<0.01	0,67	0	0	0	0	0	<0.01	0	<0.01	0,18	0,01
**Incertae Sedis XV**	0	0	0	0	0	0	0	0	0	0	0	0	0	<0.01	0	0,01
**Kordiimonadaceae**	0	0	0	<0.01	0	0,02	0	0	0	0	0	0	0,01	<0.01	0	0,02
**Lachnospiraceae**	0,02	0	0	0	<0.01	0,01	0	<0.01	0	0,01	0	0	0	0,01	0	<0.01
**Lactobacillaceae**	0	0	0	0	0	0	0	0	0	0	0	0	0	<0.01	0,03	0

**Legionellaceae**	0	0	0	0	0	<0.01	0	0	<0.01	0	0	0	<0.01	0	<0.01	0
**Lentisphaeraceae**	0	0	0	0,03	0,01	0,1	0	0	0	0	0	0	0,04	0,04	<0.01	0,01
**Leptospiraceae**	0	0	0	0	0	0	0	0	0	0	0	0	<0.01	<0.01	0	0
**Leuconostocaceae**	0	0	0	0	0	0	0	0	0	0	0	0	0	0	<0.01	0
**Methylobacteriaceae**	0	0	0	<0.01	<0.01	0	0	<0.01	<0.01	0	0	0	0,05	<0.01	0,01	0,02
**Methylococcaceae**	0	0	0	<0.01	0	0,01	0	0	0	0	0	0	0	<0.01	0	0
**Methylocystaceae**	0	0	0	<0.01	0	<0.01	0	0	0	0	0	0	0	<0.01	0	0
**Methylophilaceae**	0	0	0	0,05	0,03	0,08	0	<0.01	0	0	0	<0.01	<0.01	0	0	<0.01
**Mitochondria**	0	0	0	0	0	0	0	0	0	<0.01	0	0	0,02	0	<0.01	<0.01
**Moraxellaceae**	0,03	<0.01	<0.01	0,67	0,26	1,28	<0.01	<0.01	0,02	0,01	0,01	<0.01	0,03	0,02	0,58	0,02
**Moritellaceae**	0	0	0	0,02	0	0	0	0	0	0	0	0,02	0	<0.01	0	<0.01
**Mycoplasmataceae**	0	0	0	0	0	<0.01	0	0	0	0	0	0	0,06	<0.01	<0.01	0
**Nannocystineae**	0	<0.01	0	0	0,01	0,06	0	<0.01	0	0	0	0	0,04	0,05	0,02	0,05
**Neisseriaceae**	0	<0.01	0	<0.01	0	0	0	<0.01	<0.01	0	0	0	0	0	0,03	0
**Nitrospinaceae**	0	0,02	<0.01	0	<0.01	<0.01	0	0	0	0	<0.01	<0.01	<0.01	<0.01	0	0
**Nitrospiraceae**	2,39	2,96	2,29	2,11	0,66	0,17	0	0,02	<0.01	0,83	3,44	0,44	0	0,01	0	<0.01
**Oceanospirillaceae**	0	0	0	0,42	0	0,06	<0.01	0	<0.01	0	0	0	0,03	0,02	0,02	<0.01
**Opitutaceae**	0	0	<0.01	0,13	0,07	0,58	0,04	0,08	0,06	0,01	<0.01	0	0,93	1,13	0,67	1,12
**Oxalobacteraceae**	0	0	0	0,03	0,02	0,02	0	0	0	<0.01	0	0	<0.01	<0.01	0,04	0
**Paenibacillaceae**	0	0	0	0	0	0,01	0	0	0	0	0	0	0,01	0	0	<0.01
**Parvularculaceae**	0	0	0	0	0	0	0,18	0,02	0,04	0	0	0	<0.01	<0.01	0	0
**Pasteurellaceae**	0	0	<0.01	<0.01	<0.01	0,08	<0.01	0	0	0	0	0	<0.01	0,02	0,03	0,02
**Peptococcaceae**	0	0	0	0	0	0	0	0	0	0	0	0	0	<0.01	0	0
**Peptostreptococcaceae**	0	0	0	0	0	0	0	0	0	0	0	0	<0.01	<0.01	0	0
**Phyllobacteriaceae**	0,94	0,53	0,15	0,51	1	0,05	<0.01	0,03	0	0,57	1,23	0,36	0,04	0,02	0,06	0,03
**Piscirickettsiaceae**	0	<0.01	<0.01	<0.01	0	0,04	<0.01	0	0	0	<0.01	<0.01	0,03	0,02	0,02	0,03
**Planctomycetaceae**	0	0,01	<0.01	0,07	0,12	0,47	0,01	<0.01	0,01	0,01	<0.01	<0.01	0,43	0,33	0,42	0,41
**Planococcaceae**	0	0	0	0	0,01	0,03	0	<0.01	0	0	0	0	0	0	0	0
**Porphyromonadaceae**	0	0	0	0	0	<0.01	0	0	0	0	0	0	0	0	0	0
**Prevotellaceae**	0	0	0	0	0	0	0	0	0	0	<0.01	0	0	<0.01	0,01	0
**Pseudoalteromonadaceae**	0	0	0	6,47	0,07	0,41	0	0,01	0	0	0	<0.01	0,08	0,02	0,14	0,07

**Pseudomonadaceae**	0	0,01	0,01	0,06	0,04	0,54	0	<0.01	<0.01	0,03	0,36	0,03	0,43	0,01	15,4	0,25
**Psychromonadaceae**	0	0	0	<0.01	0	0	0	0	0	0	0	0	0	<0.01	0,01	0
**Rhizobiaceae**	0	0,01	0	0	0,01	0,01	0	<0.01	0	0	0	0	<0.01	<0.01	<0.01	0
**Rhodobacteraceae**	0,1	0,07	0,18	2,18	3,83	3,91	0,96	0,68	1,03	4,95	2,69	1,03	8,4	9,37	4,53	11,5
**Rhodobiaceae**	0	<0.01	0	0	<0.01	0,04	0	0,01	0	0,07	0	<0.01	<0.01	0	0,02	<0.01
**Rhodocyclaceae**	0,01	0	0	0,17	0,04	0,12	0,02	0,03	<0.01	0	0	0	0,02	<0.01	0,01	<0.01
**Rhodospirillaceae**	0	0	0	0,02	0,02	0,01	0	0	0	0,05	0	0	0,01	<0.01	0	0,02
**Rickettsiaceae**	0	0	0	<0.01	0	0	0	0	0	0	0	0	<0.01	<0.01	0	<0.01
**Rikenellaceae**	0	<0.01	0	0	0	0	0	0	0	0	0	0	0,02	<0.01	0,01	<0.01
**Rubrobacterales**	0,1	0,07	0,23	0,04	0,03	0	0	0,01	0	0	0,02	0	0	<0.01	<0.01	0
**Ruminococcaceae**	0	<0.01	0	0	0	0	<0.01	0	0	0	0	<0.01	0,01	0,01	<0.01	<0.01
**Saccharospirillaceae**	0	0	0	<0.01	0	0	0	0	0	0	0	0	0,01	<0.01	0,01	0
**Salinisphaeraceae**	0	0	0	0,01	0,02	0,01	0	0	0	0	0	0	0	<0.01	0	0
**Saprospiraceae**	0	0	0	0,07	0,02	0,17	<0.01	0,01	0,03	0	0	0	0,12	0,19	0,1	0,2
**Shewanellaceae**	0	<0.01	<0.01	0,14	0,02	0,04	0	0	0	0	0	0	0,02	0,01	0	0,01
**Simkaniaceae**	0	0	0	0	0,02	<0.01	0	0	0	0	0	0	<0.01	<0.01	0	0
**Sorangineae**	0	<0.01	0	<0.01	0,02	<0.01	0	0,01	0,02	<0.01	0	0	0,05	0,01	0,01	0,03
**Sphingomonadaceae**	0,04	<0.01	0,04	0,63	0,09	1,09	<0.01	0,07	0,02	0,03	0,01	0,04	0,95	0,25	0,3	0,14
**Spirochaetaceae**	0	0	0	0	0	0	0	0	0	0	0	0	0	<0.01	<0.01	<0.01
**Sporolactobacillaceae**	0	0	0	0	0	<0.01	0	0	0	0	0	0	0	0	0	0
**Staphylococcaceae**	0	<0.01	0,01	0,09	0,01	0,02	0	0,01	0	0	0	0	0	<0.01	0,02	<0.01
**Streptococcaceae**	0	<0.01	0	0,03	0,03	<0.01	0	0	0	0,01	0	0,01	<0.01	<0.01	0,06	0
**Subdivision 5**	0	0	0	0	<0.01	<0.01	<0.01	0	0	0	0	0	0,01	0,02	0,02	0,02
**Syntrophaceae**	0,22	0,72	0,11	0,16	0,3	<0.01	0	0,03	<0.01	0,48	0,19	0,1	<0.01	<0.01	0	<0.01
**Syntrophobacteraceae**	0	0	0	0	0	0	0	0	0	0	0	0	0	<0.01	0	0
**Syntrophomonadaceae**	0	0	0	0	0	<0.01	0	0	0	0	0	0	0	0	0	0
**Thermaceae**	0	0	0	0	0	0	0	0	0	0	0	<0.01	0	0	0	0
**Thermoactinomycetaceae**	0	0	0	0	0	0	<0.01	0	0	0	0	0	0	0	0	0
**Thermotogaceae**	0	<0.01	<0.01	0	0	0	0	0	0	0	0	0	0	0	0	0
**Thiotrichaceae**	0	0	0	0,02	0,02	0,01	0	0	0	0	0	0	0	<0.01	0	0
**Trueperaceae**	0	0	0	<0.01	0	0,02	0	0	0	0	0	0	<0.01	<0.01	0	0

**Verrucomicrobiaceae**	0,01	0	0,02	0,03	0,02	0,14	0,03	0	0,02	0	0	0	0,26	0,3	0,09	0,41
**Vibrionaceae**	0	0	0	0,2	0,07	0,31	0	0	<0.01	0	0	0,03	0,15	0,17	0,14	0,17
**Victivallaceae**	0	0	0	0	0	0	0	0	0	0	0	0	0	<0.01	0	0
**Xanthomonadaceae**	0,07	0,3	0,32	0,15	0,36	0,17	0	0,19	<0.01	<0.01	0,1	0,04	<0.01	0,01	0,19	0,02
**Xiphinematobacteriaceae**	0	0	0	0	<0.01	0,02	<0.01	0	0	0	0	0	<0.01	<0.01	0,01	0,02
**family_BTH**	14,6	18,1	14,7	8,08	17,8	7,19	40,8	31,2	17,9	13,2	20,4	15,6	5,48	4,71	4,06	5,21
**family_NA**	52,9	51,4	52,7	23,8	43,3	8,6	49,4	57,3	73,4	50,7	49,9	61,7	31	32,5	25,8	33
**uncl_"Thermoanaerobact."**	0	0	0	0	0	0	0	0	0	0	0	0	0	<0.01	0	0
**uncl_Actinobacteridae**	0	0	0	0	0	<0.01	0	0	0	0	0	0	<0.01	<0.01	0	<0.01
**unclassified_Bacillales**	0	0	0	0	0	<0.01	0	0	0	0	0	0	0	0	0	0
**unclassified_Bacteroidales**	0	0	0	0	0	<0.01	0	0	0	0	0	0	<0.01	0,02	0	0,03
**uncl_Burkholderiales**	0	0	0	0	0	0	0	0	<0.01	0	0	0	0	<0.01	0,01	0
**unclassified_Chlamydiales**	0	0	0	0	0	0	0	0	0	0	0	0	0	<0.01	0	0
**unclassified_Chromatiales**	2,02	1,95	2,16	0,67	1,56	0,14	0	0,02	0	0,21	0,43	0,11	0,04	0,02	0,06	0,08
**unclassified_Clostridiales**	0	0	0	0	0	<0.01	0	0	0	0	0	0	<0.01	<0.01	0	<0.01
**uncl_Desulfuromonales**	0	<0.01	0	0	0	0,01	0	0	0	0	0	0	0	<0.01	0	0
**uncl_Flavobacteriales**	0,01	<0.01	0	0,01	0,02	0,03	0,04	0,01	0,03	<0.01	0	0	0,9	2,86	0,48	1,89
**uncl_Fusobacteriales**	0	0	0	0	0	<0.01	0	0	0	0	0	0	0	0	0	0
**unclassified_Myxococcales**	0	0	0	0	0	0,04	0	0	0	0	<0.01	0	0,04	0,02	0,02	0,07
**uncl_Oceanospirillales**	5,9	4,45	4,49	1,43	2,88	0,52	0,11	0,17	0,11	3,37	3,42	4,93	0,93	1,29	0,63	1,26
**unclassified_Rhizobiales**	0	0,01	0	0,12	0,04	0,22	0,01	<0.01	0,02	0	<0.01	<0.01	0,15	0,11	0,27	0,12
**uncl_Rhodospirillales**	0,04	0,08	0,05	0,15	0,06	0,14	0,25	0,13	0,17	1,17	0,09	0,06	2,17	2,06	1,75	2,21
**unclassified_Rickettsiales**	0	0	0	0	0	<0.01	5,26	5,53	3,79	<0.01	0	<0.01	<0.01	<0.01	0	0
**uncl_Sphingobacteriales**	0	0	0	<0.01	0	0	0	0	0	0	0	0	<0.01	<0.01	0	0
**uncl_Syntrophobacterales**	0	0	0	0	0	<0.01	0	0	0	0	0	0	0	<0.01	<0.01	<0.01
**uncl_Verrucomicrobiales**	0	0	0	0,04	0,05	0,12	0,01	<0.01	0,01	0	0	0	0,4	0,4	0,16	0,43

Table S4 Taxonomic assignments of V6 sequence tags from sponge and seawater samples to 'sponge-specific' 16S rRNA sequence clusters. Relative abundances (in %) are denoted. Sample numbers: 1-3, *R. odorabile*; 4-6, *R. odorabile* larvae; 7-9, *I. basta*; 10-12, *I. ramosa*; 13-16, seawater.

	**1**	**2**	**3**	**4**	**5**	**6**	**7**	**8**	**9**	**10**	**11**	**12**	**13**	**14**	**15**	**16**
**SC 1 (Cyanobacteria)**	0	<0.01	0	<0.01	0	0	0	0.05	0	0.13	0.14	0.12	0	<0.01	0	<0.01
**SC 3 (Cyanobacteria)**	0	0	0	0	0	0	0	0	0	0	0	0	<0.01	<0.01	0	<0.01
**SC 7 (Chloroflexi)**	0.03	0.07	0.05	0.02	0.05	0.01	0	0	0	0	<0.01	0	0	0	0	0
**SC 8 (Chloroflexi)**	0.03	0.11	0.04	0.02	0.04	<0.01	0	0	0	0	0	0	0	0	0	0
**SC 9 (Chloroflexi)**	0.17	0.11	0.05	0.03	0.07	<0.01	0	<0.01	0	0.03	0	0	0	0	0	0
**SC 10 (Chloroflexi)**	0	0	0	0	0	<0.01	0	0	0	0	0	0	0	0	0	0
**SC 11 (Chloroflexi)**	1.07	1.19	1.3	0.51	1.31	0.12	0	<0.01	0	0.23	0.41	0.57	0	0	0	0
**SC 12 (Chloroflexi)**	2.6	2.8	2.26	0.41	0.48	0.11	0.01	0.02	0	0.14	0	0	0	<0.01	0	0
**SC 13 (Chloroflexi)**	4.17	4.46	5.04	1	1.77	0.17	0	0.06	0	0.49	0.61	<0.01	<0.01	0	<0.01	0
**SC 14 (Chloroflexi)**	0.11	0.04	0.08	0	0.11	<0.01	0	<0.01	0	<0.01	0.07	0.05	0	0	0	0
**SC 15 (Chloroflexi)**	0.21	0.16	0.29	0.11	0.23	0.08	0	0.02	0	0.29	0.56	0.46	<0.01	0	0	0
**SC 16 (Chloroflexi)**	0.81	0.41	0.62	0.99	2.15	0.3	0	0.05	0.01	1.11	2.18	1.75	0	<0.01	0	0
**SC 17 (Chloroflexi)**	1.28	1.04	0.81	0.44	0.54	0.06	0	0.11	0	1.48	2.02	1.56	0	<0.01	0	0
**SC 19 (Acidobacteria)**	0.58	0.98	0.56	0.09	0.14	0.06	0	0.03	0	0.12	0.14	0	0	<0.01	0	0
**SC 20 (Deltaproteobacteria)**	0.84	1.41	1.04	0.06	0.11	0.03	0	0.03	0	0.17	<0.01	0	0	0	0	0
**SC 22 (Gemmatimonadetes)**	1.68	1.73	0.94	1.95	4.46	0.2	0	0.1	0.02	1.82	0.61	0.82	0.02	<0.01	0	<0.01
**SC 23 (Nitrospirae)**	2.38	2.96	2.29	2.11	0.66	0.17	0	0.02	<0.01	0.83	3.44	0.44	0	<0.01	0	0
**SC 25 (Poribacteria)**	4.08	3.28	4.24	1.17	3.5	0.27	0	0.06	0.01	1.45	3.08	2.01	0	0	0.02	0
**SC 26 (putative new phylum)**	4.38	2.21	5.15	0.94	2.57	0.21	0	0.12	<0.01	3.31	3.47	6.25	0	<0.01	<0.01	<0.01
**SC 28 (Deltaproteobacteria)**	1.23	0.84	0.73	0.83	1.93	0.17	<0.01	0.08	<0.01	4.69	3.13	5.56	0	<0.01	0	0.01
**SC 29 (Spirochaetes)**	0.07	0.21	0.14	0.02	0.08	<0.01	0	<0.01	0	0.02	0.08	<0.01	0	0	0	0
**SC 32 (Actinobacteria)**	1.47	1.75	2.8	2.09	4.32	0.38	0	0.11	0.01	2.43	0.79	8.73	0	<0.01	0	0
**SC 34 (Actinobacteria)**	0.01	<0.01	0	0	<0.01	0	0	0	0	0	0	0.01	0	0	0	0
**SC 35 (Actinobacteria)**	0.04	0.08	0.5	0.1	0.13	0.05	0	<0.01	0	0.07	1.22	0.51	0	0	0	0
**SC 48 (Alphaproteobacteria)**	0	<0.01	0	0	0	0	0	0	0	0	<0.01	0	0	0	0	0
**SC 51 (Alphaproteobacteria)**	0.18	0.85	0.27	0.26	0.42	0.1	0	0.03	0	1.31	0.22	0.15	0	0	0	0

**SC 52 (Alphaproteobacteria)**	0	0	0	0	0	<0.01	0	0	0	0	0	0	0	0	0	0
**SC 53 (Alphaproteobacteria)**	0.42	0.39	0.31	1.28	1.27	0.07	0	0.03	0	2.09	2.02	1.88	0	0	0	0
**SC 54 (Gammaproteobacteria)**	0	0	0	0	0	0	0	0	0	0	0	0	<0.01	0	0	0
**SC 59 (Gammaproteobacteria)**	0	0.09	0.09	0.04	0.2	<0.01	0	0.05	0	<0.01	1.03	0.05	0	0	0	0
**SC 60 (Gammaproteobacteria)**	4.22	3.42	3.11	1.15	1.9	0.1	<0.01	0.04	0	1.18	1.11	0.19	0.02	<0.01	<0.01	0.01
**SC 63 (Gammaproteobacteria)**	0.67	0.59	0.31	0.14	0.29	0.05	0	0.02	0	0.29	0.18	0.12	0	0	0	0
**SC 69 (Gammaproteobacteria)**	0	0	0	0	0	0	0	0	0	0	0	0	<0.01	0	0	0
**SCC A (Acidobacteria)**	0.04	0.17	0	0.1	0.33	<0.01	0	<0.01	0	0.85	0.18	0.93	0	0	0	<0.01
**SCC B (Acidobacteria)**	2.01	1.88	1.52	0.47	0.39	0.11	0.01	0.12	0	0.53	0.2	0.18	0	<0.01	0	0
**SCC C (Acidobacteria)**	1.76	0.89	0.93	0.46	0.89	0.77	0	0.28	0.02	7.96	2.45	2.4	0	0	0.01	0
**SCC D (Deltaproteobacteria)**	0.71	1.12	1.26	0.65	1.34	0.1	0.02	0.01	0	0.27	0.22	0.52	0	<0.01	0	0.01
**SCC E (Gemmatimonadetes)**	2.59	3.78	3.94	1.43	2.42	0.45	0	0.2	<0.01	5.84	3.91	4.12	<0.01	0	0.02	0.01
**SCC F (Nitrospirae)**	0	0	0	0	0	0	0	0	0	0	<0.01	0	0	0	0	0
